# Large‐scale correlations between gamebird release and management and animal biodiversity metrics in lowland Great Britain

**DOI:** 10.1002/ece3.10059

**Published:** 2023-05-08

**Authors:** Joah Robert Madden, Rosie Buckley, Sophia Ratcliffe

**Affiliations:** ^1^ Psychology, Centre for Research in Animal Behaviour University of Exeter Exeter UK; ^2^ NBN Trust Nottingham UK

**Keywords:** farmland birds, generalist predators, mallard, partridge, pheasant, shooting, woodland birds

## Abstract

The ecological effects on populations of non‐game species driven by the annual release and management of tens of millions of gamebirds for recreational shooting are complex and relatively poorly understood. We investigated these effects at a national scale, considering multiple taxa simultaneously. We used records from the UK National Biodiversity Network Atlas to compare animal species and diversity metrics previously suggested to be affected by behaviors of the released birds, or because resources or habitats are influenced by game management or both processes. We contrasted records from 1 km grid squares where gamebirds were reported released in Great Britain, and control squares with similar land cover but where no releases were reported. There were more records overall reported from release grid squares (RGS) compared with controls (CGS), perhaps due to greater reporting effort or greater biological richness. We found fewer foxes in RGS and fewest in grid squares with largest releases, but more carrion crows in RGS. We found no consistent effects for buzzards, ravens, jays, or magpies. There were more rodents and gray squirrels reported from RGS but no differences for reptiles. There were more butterflies but fewer beetles reported from RGS but no consistent patterns for Orthoptera or ground beetles considered common gamebird prey. Farmland and woodland birds exhibited higher abundance, richness, and diversity in RGS when considering absolute records, but woodland bird abundance and richness were lower when correcting for the relative number of records. These nationwide results, despite crude data resolution, reveal diverse effects of gamebird release and management at a national scale and across trophic levels, increasing some non‐game animal populations while decreasing others. This should alert practitioners, opponents, and legislators that a focus on single taxa effects, either positive or negative, may obscure the simultaneous changes in other taxa.

## Graphical abstract

The release of gamebirds in the GB is accompanied by changes in populations of non‐game species in those areas where release and management occur. These changes include both increases and decreases and span a wide range of taxa and trophic levels. The ecological effects of the release and subsequent management of gamebirds in GB are complex and concentrating on changes in single taxa may miss other, simultaneous, effects.

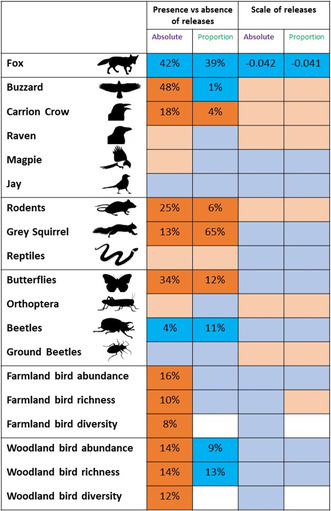

## 1 INTRODUCTION

Recreational shooting is supported by the annual release and subsequent management of tens of millions of gamebirds across millions of hectares of agricultural, grassland, or woodland; a practice common across Europe, North America, and New Zealand. In the UK, where gamebird release and management is long‐established and widespread, an estimated 32 million pheasants *Phasianus colchicus*, 9 million red‐legged partridges *Alectoris rufa*, and 3 million mallard *Anas platyrhynchos* are released annually (95% CI of total releases 29.0–57.3 million; Madden, 2021) while other estimates put the figure at 57 million (range 47.1–70.0 million; Aebischer, 2019). Their release motivates management action including habitat creation or retention, supplementary feeding, and predator control (Sage et al., 2020). In the UK, this involves ~90,000 km^2^ of lowlands (PACEC, 2014), including 14%–28% of woodland (Gilbert, 2007). At the time of release, in later summer, these birds may constitute ~45% of the total bird biomass in the UK (Blackburn & Gaston, 2021).

This release and management activity exerts a series of ecological effects on the local fauna and flora with a range of implications for the conservation of particular species, suites of species, or ecological networks (Reviewed by Madden & Sage, 2020; Mason et al., 2020; Sage et al., 2020). The net ecological consequences are likely to be complex: The birds themselves exert direct effects (e.g., eating plants and animals; altering nutrient levels; acting as disease vectors; directly competing with local species; supplementing the diet of predators); the management to support them post‐release by gamekeepers and landowners exerts associated effects (e.g., habitat creation or retention, predator control, supplementary feeding), and these direct and associated effects have indirect effects via ecological networks on other wildlife and habitats (Madden & Sage, 2020). This network of ecological interactions can make determining overall effects difficult. Previous work has suggested that particular taxa may be especially sensitive to these activities, but the direction and magnitude of the effects is contentious, with empirical studies and hypothesized mechanisms supporting both increases and decreases (reviewed by Madden & Sage, 2020; Mason et al., 2020; Sage et al., 2020). Some specific examples include: avian predators Pringle et al., 2019; invertebrates Hall et al., 2021; Woodland birds Draycott et al., 2008; vegetation Sage et al., 2009).

Generalist predators and scavengers including foxes *Vulpes vulpes*, Buzzards *Buteo buteo* and corvids (e.g., carrion crows *Corvus corone*, magpies *Pica pica*, and ravens *Corvus corax*) might be expected to thrive, eating the (carcasses of) released birds, causing increases in the predator populations, or local abundances (Lees et al., 2013; Pringle et al., 2019; Swan et al., 2022). Alternatively, their numbers may be constrained or reduced due to legal or illegal control by gamekeepers (Heydon et al., 2000; Porteus et al., 2019). Populations of small quadrupeds including rodents, gray squirrels *Sciurus carolinensis*, and reptiles may all increase due to the provision of supplementary food, the creation and management of suitable habitats, the reduction in predators, or reduced human disturbance (e.g. Davey, 2008; Saad et al., 2021; Sanchez‐Garcia et al., 2015). Alternatively, populations may decline due to direct predation by the released gamebirds themselves, competition for resources from the released gamebirds, or increases in generalist predators supported by supplementary gamebird prey (e.g. Davey, 2008). Invertebrate populations, predominantly insects, have been proposed to increase due to habitat creation and management or nutrient enrichment (e.g. Hall et al., 2021; Robertson et al., 1988). Conversely, populations at release sites may decline or change in composition due to direct predation if eaten by omnivorous gamebirds or because of habitat damage (e.g., Hall et al., 2021; Neumann et al., 2015; Pressland, 2009). Finally, populations of non‐game farmland and/or woodland birds may increase due to supplementary feeding, the provision or management of improved habitats, or reduced disturbance (e.g., Davey, 2008; Hoodless et al., 2006; Robertson et al., 1988; Sage et al., 2005, 2009). Alternatively, such populations might decline through competition for resources by released gamebirds, increases in generalist predators, or exposure to diseases carried by gamebirds (e.g., Bicknell et al., 2010; Gethings et al., 2016; Tompkins et al., 2000).

This ambiguity in effects of gamebird release and management may arise because our current understanding may be based on studies conducted at just one or a few sites (because of the logistical costs of ecological sampling; but see examples with >10 sites e.g. Davey, 2008; Draycott et al., 2008; Hall et al., 2021; Neumann et al., 2015; Pressland, 2009; Sage et al., 2009) or involve the voluntary participation of shoot owners (presenting an opportunity for sampling bias with more environmentally aware shoot owners opting to host research; e.g. Cox et al., 1996; Howard & Carroll, 2001; Saad et al., 2021; Sanchez‐Garcia et al., 2015). Effects may vary spatially and/or temporarily, which is problematic if the study focus is on a restricted region or lasts only one or a few seasons (e.g. Hall et al., 2021; Heydon et al., 2000; Neumann et al., 2015; Woodburn & Sage, 2005). An alternative approach has been to conduct a correlative study between nationwide records collected within grid squares. For example, Corke (1989) reported that UK 10 km^2^ grid squares containing pheasants were less likely to have certain butterflies present. Pringle et al. (2019) analyzed the relationships between reared gamebird metrics and avian predator numbers at the resolution of 10 km^2^ grid squares. One problem with analyses at this scale (in addition to the fact that such correlations may not be causal: see Robertson et al., 1988) is that gamebird releases are often highly localized. Most shooting estates typically cover <400 ha and the majority of released pheasants typically disperse <1 km (Madden & Sage, 2020).

To better understand the ecological effects of gamebird release and management on non‐game animal populations, we should explore relatively fine‐scale spatial associations between gamebird release and management with records of taxa of interest across a large area. Here, we do this by combining official records of gamebird release with a national database of UK biodiversity records. We analyze datasets at the level of 1 km^2^ grid squares and this allows us to compare biodiversity records in grid squares where release occurs (Release Grid Squares—RGS) with control grid squares (CGS), drawn from a sample that matches the land cover composition seen at release sites. We also look within RGS for relationships between the numbers of gamebirds reported as released and biodiversity records. This allows us to ask, at a national scale, how does the presence and scale of gamebird release and management relate to populations of non‐game fauna in the UK?

## 2 METHODS

### 2.1 Release data

**FIGURE 1:**
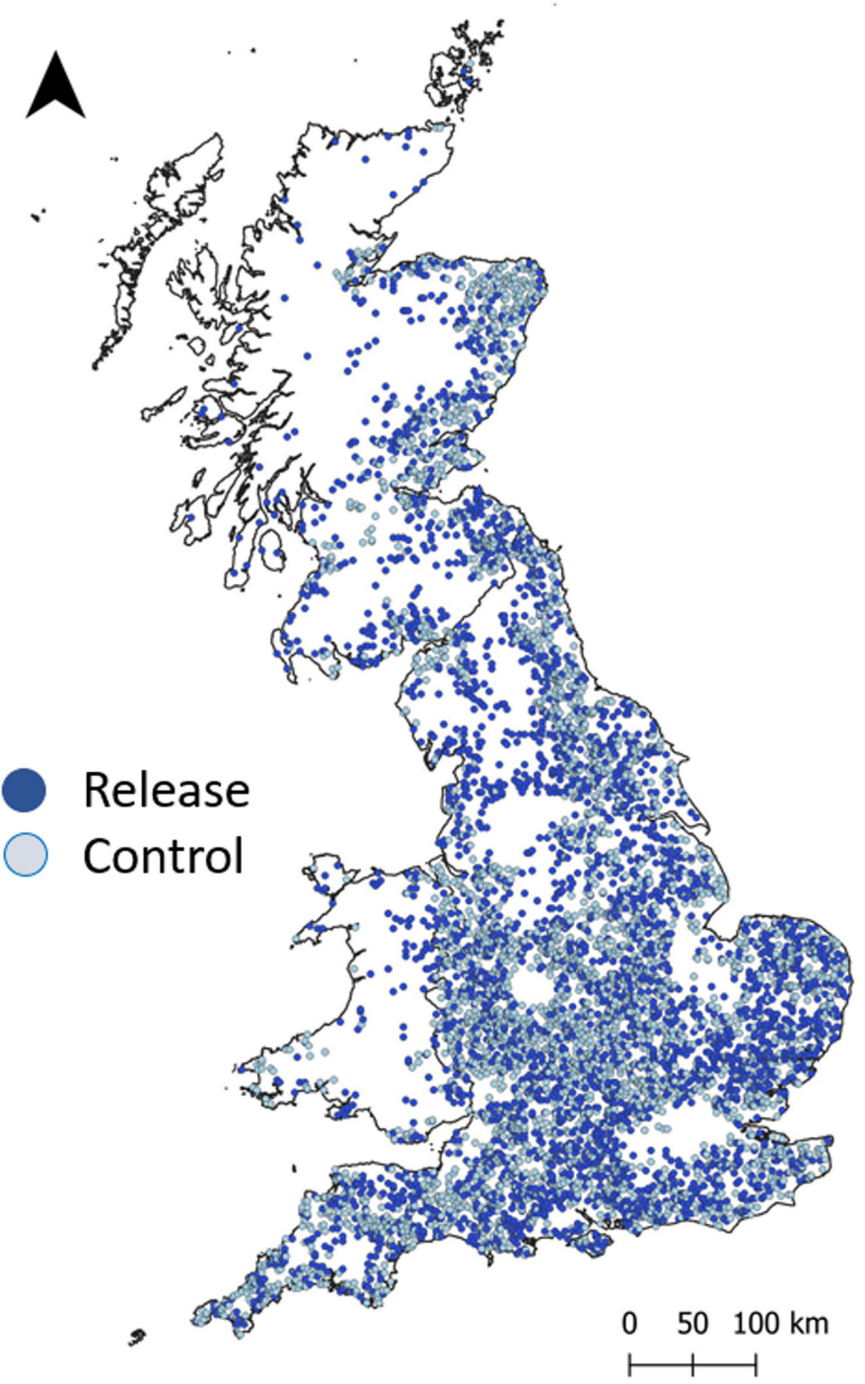
Release (dark) and Control (light) grid squares used in this study shown at 5 km resolution. Control grid squares were drawn from a set of grid squares that had similar land‐cover composition to grid squares where releases were reported.

A registration system, the APHA Poultry Register is obligatory for holdings with flocks of more than 50 birds and voluntary registration of flocks that are smaller than 50 birds is encouraged. We engaged in a Data Sharing Agreement with the APHA in conjunction with Natural England and received the data on 10 Feb 2021. We filtered the data so that it only included gamebird species (pheasant, partridge; no separation of red‐legged and gray) and duck (no distinction by species) and numbers held for release (as opposed to those registered held for breeding or rearing) as denoted under Livestock Unit Animal Purpose as release for shooting. This included details of Usual Stock Numbers of gamebirds designated as held for release at 3624 sites in the UK. We removed sites where Usual Stock Numbers were zero, grid squares with >50% urban cover (where we assumed that the person completing the Register had mistakenly given their home address rather than the site where the birds were held for release), and sites in Northern Ireland (not included in the Centre for Ecology and Hydrology [CEH] land cover data—see below). When a 1 km^2^ grid square contained >1 release site, we combined Usual Stock Numbers for all sites in the grid square. This left us with 3284 grid squares where gamebirds were reported as being held for release which we defined as Release Grid Squares (RGS) (Figure 1).

Although registration with the Poultry Register is a legal requirement for holdings of ≥50 birds, it appears that compliance is low with the register only accounting for about one‐third of the gamebirds estimated to be released in the UK annually and about half to one‐third of the number of locations compared to the estimated number of shoots releasing gamebirds (Madden, 2021). Therefore, many of our control sites, defined by an absence of records in the poultry register, may host shoots or releases. This means that detecting differences between sites (definitely) with and (apparently) without releases becomes somewhat obscured and subtle differences may be missed. Therefore, our approach is a conservative one and any differences that are detected are likely to be meaningful rather than spurious. We attempted various ways to assess whether putative control sites may actually host shoots (see Validating functional differences between release and control grid squares below) but although we could suggest some overall levels of misidentification, we could not accurately confirm or reject a site as definitely not hosting released gamebirds without a field site inspection visit to all 3000+ squares which was beyond the capacity of this project.

### 2.2 Selection of control grid squares

We derived a set of Control Grid Squares (CGS) by ensuring that they contained similar land cover patterns to those seen in RGS because it would be uninformative to compare biodiversity measures from RGS which are reported to mainly occur in lowland agricultural areas with CGS in the uplands, urban areas or coastal sites where the species compositions are going to be markedly different. Therefore, we used the CEH land cover data 2015 to examine the landcover measures in grid squares. The aggregated CEH data provides a percentage cover of 10 different habitat types for each 1 km^2^ of Great Britain (Rowland et al., 2017). The data were filtered to remove all sites with a total 0 cover (1 km squares found at sea).

First, we compared RGS with all other grid squares in the UK (Appendix S3). As expected, RGS contained more arable, broadleaf woodland and improved grassland and less built‐up, coastal, conifer woodland, montane/bog, semi‐natural grassland, and saltwater cover than an average area in the UK. This confirms that the land cover in grid squares where gamebirds are released is of a particular type. Second, we sought grid squares that contained a similar mix of habitats to RGS but where no releases were reported. We compiled a set of grid squares where no birds were reported as being held for release and whose land cover for each aggregate class was within 1SD of the mean habitat cover percentage of RGS. This produced a pool of 32,147 grid squares. We drew a random set of 3284 grid squares from this initial set. Finally, we visualized our sets of grid squares to inspect whether they appeared to occur in areas that we expected to be suitable for releasing and shooting gamebirds (Figure 1).

For RGS, we included data on the number of gamebirds (combined across all three species) reported as held for release. This varied across the grid squares from 1 to 255,500 birds/site. The distribution of releases was highly skewed. Most sites (2465; 75%) reported releases of less than 3000 birds. These could be classified as small shoots (see Madden, 2021 for definitions of shoot classes). Only 309 sites (9%) reported releasing more than 10,000 birds, being classified as large shoots.

### 2.3 Biodiversity data

Observational wildlife records for release sites and corresponding controls were extracted from the NBN (National Biodiversity Network) Atlas (NBN Atlas, ND). NBN Atlas provides records of species in the UK, Channel Islands, and Isle of Man, compiled from multiple different data providers (See Appendices S1 and S2). We extracted records that were available at a minimum precision of 1 km with no interpolation and filtered them to only contain observations from 2000 to 2020. For each grid reference, the total number of observations was extracted. No records were reported from 267 CGS and 266 RGS (8% each) and more than 100 records were submitted from 1000 of CGS and 1200 of RGS (30% and 37%, respectively). In total, there were 1,081,248 records across all surveyed taxa from CGS and 1,432,318 records from RGS. We included 69 taxa in our analyses (Table 1). For indicator farmland and woodland bird species, we calculated, for each grid square, the total abundance, species richness, and Shannon diversity index.

**TABLE 1:** Taxa of interest used in our analyses and the inclusion terms from the NBN Atlas used to extract their records.

Taxa of interest	Taxa included (inclusion terms in NBN atlas)
Gamebirds	*Phasianus colchicus*, *Alectoris rufa*, *Anas platyrhynchos*
Generalist predators	*Vulpes vulpes*, *Buteo buteo*, *Pica pica*, *Corvus corax*, *Corvus corone*
Rodents	Order: Rodentia
Gray squirrel	*Sciurus carolinensis*
Reptiles	Class: Reptilia
Day‐time flying butterflies	Family: Papilionidae, Hesperiidae, Pieridae, Nymphalidae, Riodinidae, Lycaenidae
Beetles	Order: Coleoptera
Ground beetles	Family: Carabidae
Grasshoppers	Order: Orthoptera
Farmland birds	*Passer montanus*, *Streptopelia turtur*, *Perdix perdix*, *Motacilla flava*, *Sturnus vulgaris*, *Linaria cannabina*, *Vanellus vanellus*, *Emberiza citronella*, *Alauda arvensis*, *Falco tinnunculus*, *Emberiza schoeniclus*, *Curruca communis*, *Chloris chloris*, *Corvus frugilegus*, *Columba oenas*, *Carduelis carduelis*, *Columba palumbus*, *Coloeus monedula*
Woodland birds	*Turdus merula*, *Cyanistes caeruleus*, *Pyrrhula pyrrhula*, *Fringilla coelebs*, *Prunella modularis*, *Parus major*, *Curruca curruca*, *Aegithalos caudatus*, *Erithacus rubecula*, *Turdus philomelos*, *Strix aluco*, *Troglodytes troglodytes*, *Sylvia atricapilla*, *Phylloscopus collybita*, *Periparus ater*, *Sylvia borin*, *Regulus regulus*, *Dendrocopos major*, *Picus viridis*, *Garrulus glandarius*, *Dryobates minor*, *Poecile palustris*, *Luscinia megarhynchos*, *Sitta europaea*, *Acanthis cabaret*, *Phoenicurus phoenicurus*, *Accipiter nisus*, *Muscicapa striata*, *Phylloscopus sibilatrix*, *Spinus spinus*, *Anthus trivialis*

### 2.4 Validating functional differences between release and control grid squares

We explored how well our classification of grid squares captured the scale of gamebird release and management. We compared the total records of gamebird species reported in RGS and CGS. Overall, there were 68% more records from RGS (*x* = 3.59) than CGS (*x* = 2.14) (Mann–Whitney *U*‐test *U* = 5,092,721, *n*_Release_ = *n*_Control_ = 3284, *p* < .0001). We also tested how well the grid square classification reflected the extent of habitat management associated with game releasing and shooting. We randomly selected 100 RGS and CGS and viewed them on Google Earth (with grid squares demarked using http://nearby.org.uk/google.html#9) using the Historic View function with images dating back to 2000. Each grid square was viewed by JRM, blind to the classification, known to RB. The number of game strips in each grid square was counted. Game strips are typically planted along field margins or woodland edges and comprise thin areas of mixes of vegetation that contrast with the crops of grassland in the adjoining field, but they are often not separated from those crops by a hedge (Game Conservancy Limited, 1994). Such strips may have tracks cut through them to encourage gamebirds to flush from them when beaten, while other strips may contain visible feeders or pens and shelters. Around one‐fifth (19%) of the CGS that we viewed contained game strips compared with about two‐fifths (39%) of the RGS. We observed more game strips in RGS (0.76/km^2^) than CGS (0.35/km^2^) (*t*_176.58_ = 2.81, *p* = .0055). These results confirm that our sets of grid squares differed in expected ways in terms of the release and management of gamebirds, but also demonstrate that at least some CGS maybe release sites that are not recorded in the Poultry Register, given the presumed relatively low compliance with the register (Madden, 2021).

### 2.5 Statistical analysis

Our data distributions for both the scale of releases and biodiversity records were highly skewed. As described above, most release sites reported relatively few birds being released with a few reporting extremely large releases. Likewise, there were relatively few biodiversity records reported from most sites compared to very detailed and intensive reporting from a smaller set of sites. We tried to take a parametric approach and construct models that would allow us to include spatial autocorrelations and habitat features and derive meaningful effect sizes. However, in the majority of our analyses, we could not get such models to converge despite trying various optimization approaches, meaning that we could not consider our models to be robust. Therefore, we decided that the appropriate, albeit cruder, way to analyze our data was to use a non‐parametric approach, comparing biodiversity measures on RGS and CGS using Mann–Whitney *U* tests and relationships between numbers of gamebird released and biodiversity measure using Spearman's correlations. Therefore, we can talk about “more or less” or “positive or negative relationships” with some confidence, but we have to treat any interpretation of effect sizes with great caution. Because median values were commonly zero, we have reported differences in mean values to give some indication of the relative differences in effects between taxa, but these should not be interpreted as biologically accurate descriptors of the magnitude of the consequences of the presence or scale of gamebird releases. For other comparisons between grid square types where data were less skewed (total biodiversity records, game strip counts), we used t‐tests assuming unequal variances. All analyses were conducted in R (Code in Appendix S4). We are not aware of formal methods to control for spatial autocorrelation where non‐paired release and control sites are distributed sporadically and compared at a national scale using non‐parametric statistics. Therefore, we conducted visual inspections of spatial depictions of all analyses for which we derived the differences between mean record numbers or bird index values from CGS and RGS pooled within 100 km^2^ tetrads (Appendix S6). We could see no clear and consistent spatial patterns in these maps, therefore we assume low/negligible spatial autocorrelation in examined effects at a national scale.

## 3 RESULTS

### 3.1 Do RGS and CGS differ in the total records of biodiversity reported?

There were 32.5% more records reported from RGS (*x* = 436) compared to CGS (*x* = 329) (Welch 2‐sample on logged data: *t*_6590.9_ = −4.74, *p* < .0001). We cannot determine whether this difference occurs because, despite an assumed equal sampling effort in RGS and CGS, there is genuinely more biodiversity at release sites, or because there were higher biodiversity sampling efforts made on RGS. We discuss the likelihood of each explanation below, but in our subsequent analyses, we consider both possibilities. We analyzed absolute record data under the assumption that sampling efforts were equal and differences in reported records reflected actual differences in biodiversity amounts. We also analyzed data for each taxa of interest controlling for the total number of records reported for that grid square, meaning that we used proportion values under the assumption that there was differing sampling effort across the grid square types.

### 3.2 How does the presence and scale of gamebird release and management relate to non‐game taxa of interest?

#### 3.2.1 Generalist predators

**FIGURE 2:**
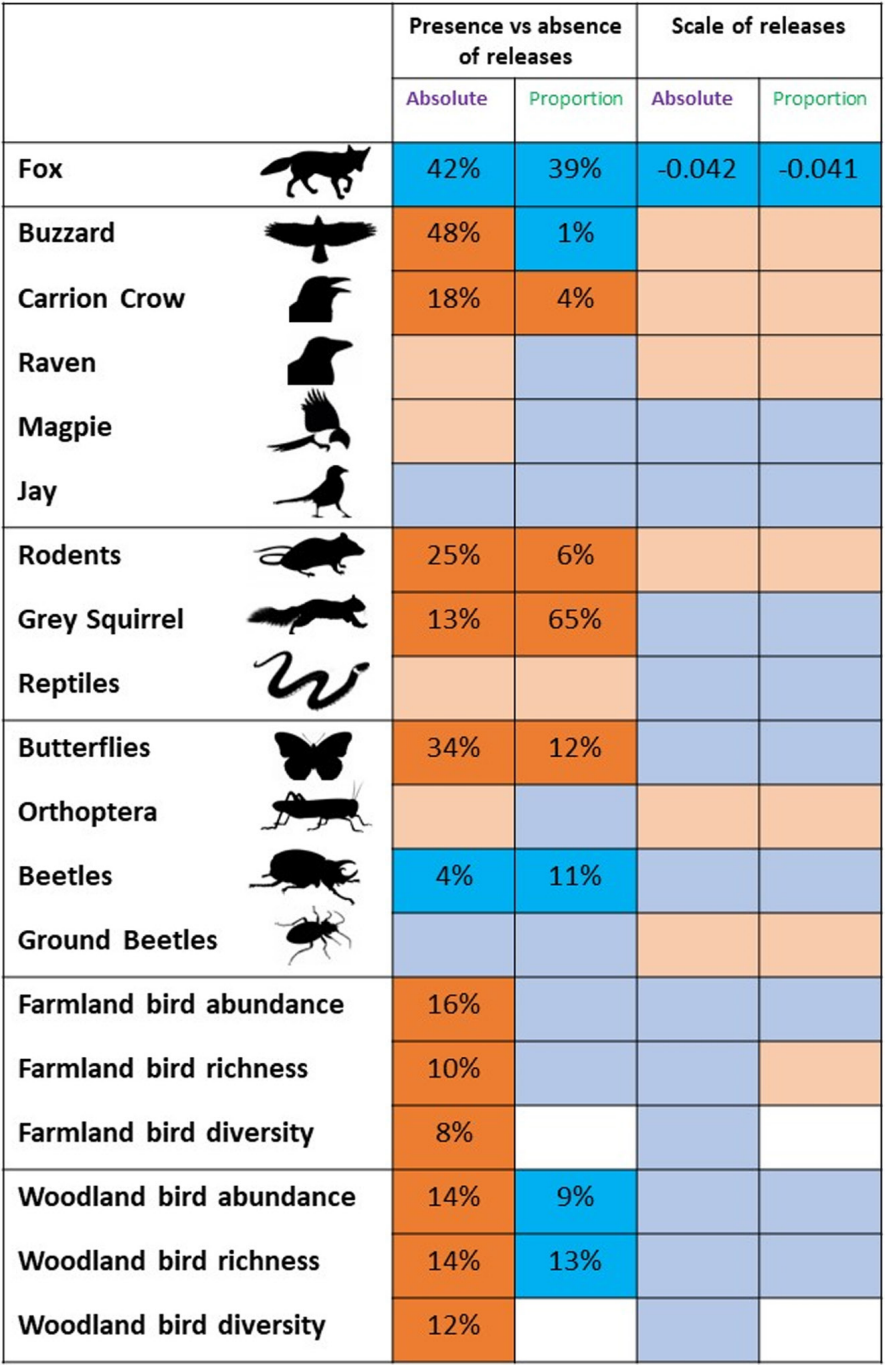
Summary of effects of gamebird release and management across a range of taxa of interest. Dark blue squares indicate that there are significantly (*p* < .05) fewer reports of the taxa of interest either on RGS compared with CGS (first pair of columns), or within RGS as numbers of birds reported as being held for release increase (second pair of columns). Light blue squares indicate non‐significant differences or relationships in the same directions. Dark orange squares indicate that there are significantly more reports of the taxa of interest either on RGS compared with CGS (first pair of columns), or within RGS as numbers of birds reported as being held for release increase (second pair of columns). Light orange squares indicate non‐significant differences or relationships in the same directions. Analyses conducted using the absolute number of records for the taxa are reported in columns 1 and 3. Analyses conducted using the relative number of records for the taxa corrected for the total number of records from the grid square, are reported in columns 2 and 4. Details of each analysis are presented in Table 2.

There were fewer foxes reported in RGS compared to CGS, and, in the only significant relationship between the size of releases and number of records for any taxa, there were fewest in RGS with largest releases when considering both absolute and relative record reports (Table 2, Figure 2). There were more carrion crows reported in RGS compared to CGS grid squares considering both absolute and relative record reports, but no relationship with the scale of releases (Table 2, Figure 2). We found no significant differences or relationships for ravens, jays, or magpies (Table 2, Figure 2). There were more buzzards reported from RGS when considering absolute record reports but fewer when considering relative record reports (Table 2, Figure 2).

**TABLE 2:** Comparisons of biodiversity records from Release Grid Squares (RGS) and Control Grid Squares (CGS) tested using Mann–Whitney *U* tests, and relationships between the numbers of gamebirds released and biodiversity records on Release Grid Squares (RGS), tested using Spearman's rank correlations.

	Raw number of records used (absolute values in Figure 2)						Records corrected for total records from the grid square (proportion values in Figure 1)					
	Mean CGS	Mean RGS	*U* (nControl = nRelease = 3284) presence vs absence values in Figure 2	*p*	Spearmans' *R* (*n* = 3284); Scale of release values in Figure 2	*p*	Mean CGS	Mean RGS	*U* (nControl = nRelease = 3284); Presence vs absence values in Figure 2	*p*	Spearmans' *R* (*n* = 3284); Scale of release values in Figure 2	*p*
Fox	0.07	0.04	5,435,070	.040	−.042	.017	0.0006	0.0003	5,435,462	.038	−.041	.018
Buzzard	0.53	0.78	5,229,047	.001	.005	.78	0.0033	0.0033	5,237,920	.003	.009	.61
Carrion Crow	1.22	1.44	5,260,290	.015	.007	.68	0.0023	0.0024	5,280,133	.039	.016	.35
Raven	0.07	0.12	5,354,530	.13	.013	.44	0.0003	0.0003	5,354,993	.13	.014	.42
Magpie	1.34	1.40	5,385,794	.90	−.020	.24	0.0016	0.0013	5,410,621	.72	−.018	.29
Jay	0.08	0.07	5,380,469	.66	−.028	.11	0.0001	0.0001	5,379,199	.63	−.028	.11
Rodents	2.21	2.76	5,191,726	<.0001	.001	.96	0.0245	0.0260	5,213,912	.001	.003	.88
Gray squirrel	0.62	0.70	5,289,708	.009	−.011	.55	0.0391	0.0064	5,293,394	.012	−.010	.57
Reptiles	0.12	0.23	5,360,335	.21	−.001	.94	0.0017	0.0035	5,361,136	.22	−.001	.94
Butterflies	5.61	7.53	5,312,055	.036	−.025	.16	0.0034	0.0038	5,312,788	.038	−.024	.16
Orthoptera	0.30	0.37	5,414,250	.54	.010	.56	0.0072	0.0045	5,415,872	.51	.012	.48
Beetles	3.01	2.89	5,197,612	.001	−.021	.23	0.0300	0.0267	5,225,840	.004	−.021	.23
Ground Beetles	0.50	0.34	5,368,864	.44	.004	.84	0.0048	0.0030	5,368,759	.43	.004	.83
Farmland bird abundance	3.03	3.50	5,245,716	.017	−.003	.86	0.0066	0.0055	5,308,180	.17	.000	.99
Farmland bird Richness	0.99	1.08	5,245,325	.017	−.005	.79	0.0057	0.0046	5,294,717	.11	.004	.81
Farmland bird diversity	0.25	0.27	5,276,437	.027	−.009	.61						
Woodland bird abundance	3.80	4.35	5,181,294	.001	−.012	.51	0.0055	0.0050	5,223,613	.006	−.007	.71
Woodland bird richness	1.46	1.67	5,182,368	.001	−.011	.52	0.0046	0.0040	5,214,683	.004	−.003	.87
Woodland bird diversity	0.32	0.36	5,225,505	.002	−.009	.61						

#### 3.2.2 Small mammals and reptiles

There were more rodents and gray squirrels reported in RGS compared to CGS when considering both absolute and relative record reports (Table 2, Figure 2). We found no significant differences or relationships for reptiles (Table 2, Figure 2).

#### 3.2.3 Invertebrates

There were more butterflies, but fewer beetles, reported in RGS compared to CGS when considering both absolute and relative record reports (Table 2, Figure 2). We found no significant differences or relationships for orthoptera or ground beetles (Table 2, Figure 2).

#### 3.2.4 Indicator birds

There were higher abundance, richness, and diversity measures based on reports of farmland and woodland birds in RGS compared to CGS when considering absolute record reports, but woodland bird abundance and richness measures were lower in RGS when considering relative record reports (Table 2, Figure 2).

## 4 DISCUSSION

Despite very crude measurement techniques and multiple sources of potential error, it is possible to detect predictable and feasible effects of gamebird release and management on a range of non‐game wildlife across Britain. As suggested previously, these effects are mixed in direction and differ across taxa with implications for a range of non‐game taxa, across trophic levels, representing both common species and those of conservation interest, either due to direct population changes or through perturbations of ecological networks.

The higher number of biodiversity reports from grid squares where gamebird releases occur demands an explanation. One explanation is that shoot owners actively encourage ecological surveys. This may be because of a high degree of engagement with environmental enhancement schemes with 77% of English gamekeepers surveyed working on shoots with a membership of an agri‐environment scheme or 36% having environmental designations on their land (Ewald & Gibbs, 2020). If this is the case, and there was simply more sampling effort conducted on RGS, then we should assume that there is no overall more biodiversity on RGS compared to CGS and instead correct for effort by looking at the relative differences in taxa records accounting for total reports from a grid square. However, this assumption of increased public surveying at release sites is not an obvious one. Instead, there is a presumption that shoots and shooting interests hamper public access (Cox et al., 1996). If this presumption is accurate, then we might expect there to be fewer reports by citizen scientists from areas where releases (and shooting) occurs because they are prevented or discouraged from surveying the area. This presumption of exclusion is not supported by surveys of landowners (admittedly made 30–40 years ago) in which owners of land with a shoot on it reported they were more likely to allow public access than average owners (Piddington, 1981) although this effect may be more pronounced on larger shooting estates (Cox et al., 1996). From a public perspective, although 17% of walkers reported that shooting had affected their use of public footpaths, this was less than half of those (39%) who reported that other obstructions (growing crops/blocked paths) affected their path use (Cox et al., 1996). Consequently, we know of little evidence that public surveys on RGS should be more difficult and hence rarer. Equally, we know of no evidence that public access to such sites is greater, explaining the higher numbers of reports. Therefore, it is reasonable to interpret the higher number of reports as indicating higher total measures of biodiversity in areas where gamebird releases occur, and we do so when considering results based on absolute numbers of reports. We also acknowledge that this may reflect real differences in biodiversity arising from active management of the habitats associated with the release of gamebirds and details of this explanation are discussed in the taxa‐specific sections below.

We found little evidence to support the contention that the release of gamebirds drives local increases in foxes (contra Roos et al., 2018) regardless of whether we considered raw or effort‐corrected records. Instead, on RGS, mean report numbers were 42% (absolute records) or 39% (relative records) lower than on CGS. Acknowledging that these effect sizes are questionable, we note that they correspond closely to modeling approaches which suggest that gamekeepers hold fox numbers at 47% of their carrying capacity on shoots (Porteus et al., 2019) and extend the observations made in at least some areas of England that fox populations are suppressed by gamekeepers (Heydon & Reynolds, 2000). The negative relationship between fox reports and the scale of releases within RGS (considering both relative and absolute records) suggests that larger shoots exert more effort in controlling foxes. This contrasts with Porteus (2015) who observed that across a sample of five shoots, larger shoots supported more foxes or exerted less effective fox control. We suggest that foxes, which in the UK can be legally killed by gamekeepers, are in at least some areas effectively suppressed, at least in the immediate vicinity of gamebird releases although these efforts may differ markedly between shoots and across regions of the UK (Heydon & Reynolds, 2000; Porteus, 2015).

We also found limited evidence to support results from a previous nationwide correlational study of relationships between gamebirds and avian generalist predators. We found higher numbers of carrion crows reported on RGS (using both absolute and relative measures). This supports Pringle et al. (2019) who found a positive relationship for winter records of crows with reared pheasants and a positive relationship between the growth rates of crow populations and gamebird biomass, although they found no relationship between their measures of released pheasants or red‐legged partridges and crow breeding population data. We found no consistent differences in numbers of ravens, jays, and magpies either between areas with and without releases or as release sizes increased. Pringle et al. (2019) found positive relationships between their measures of release pheasants and breeding and winter populations of jays and magpies and quadratic relationships with breeding ravens. The effects on buzzard populations that we detected depended on whether relative or absolute records were considered. If we interpret NBN Atlas records as reflecting real differences in biodiversity abundance then there were more buzzards reported on RGS. However, if we correct reports of buzzards for the total number of records submitted from a grid square then we conclude there are relatively fewer buzzards on RGS. Pringle et al. (2019) found a quadratic relationship between their measures of released pheasants and breeding buzzard populations and a negative relationship for winter buzzard populations. Swan et al. (2022) found a weak positive relationship between buzzard territory density and gamebird abundance during the breeding season, despite gamebirds rarely appearing in nest provisioning during that time. While crows, magpies, and jays (in England, only to conserve endangered woodland birds) can be legally controlled under general licenses in the UK, ravens and buzzards can only be legally controlled under rarely‐issued specific licenses. The higher numbers of crows on RGS suggest that gamekeepers are not effectively suppressing them, but instead, crows may be benefiting from either scavenging on dead gamebirds, access to additional resources such as supplementary food or more natural prey, or more or better habitat arising from gamebird management.

Rodents and gray squirrels appear to benefit from gamebird release and management, with higher populations on RGS. This national pattern matches some of the site‐specific findings of Davey (2008) who found that some mice and vole species were higher in woods with game management (although common shrews were rarer). However, it contradicts observations by Draycott and Hoodless (2005) that encounters with gray squirrels were no higher in woods managed for game compared to non‐game woods. Higher numbers of rodents and squirrels are most likely due to the availability of supplementary food, with effects on rat numbers corresponding to distance to these feeders even within a shoot (Saad et al., 2021). However, some small mammals may also benefit from the lower numbers of foxes, the supply of alternative prey (gamebirds) for resident predators, or the availability of suitable habitats. Despite some strong assertions that released gamebirds might predate on reptiles and so depress their populations (e.g., Milton, 2022), we found no evidence for this. Indeed there were almost twice as many records of reptiles (89% higher considering absolute records or 105% higher considering relative records) from RGS compared to CGS although due to high variance, these differences were not significant.

The mix of effects on invertebrate populations that we found resembles the variation in magnitude and direction of effects that others have reported in site‐specific studies. Neumann et al. (2015) reported 10 measures of invertebrate populations, with detected effects comprising a decrease in spring‐active or very large carabids and an increase in detritivores at high pheasant release densities, and a change in Carabidae species composition where releases occur. Other measures showed no effects of presence or scale of releasing. Hall et al. (2021) reported six measures of invertebrate populations with local decreases in total counts or biomass, but no such decreases for four focal invertebrate groups and increases for slugs and detritivores inside release pens. Devlin et al. (2021) reported eight measures of invertebrate population, with negative relations between pheasant activity and total invertebrate abundance (but not diversity) and Hymenopteran abundance, but no effects on the other five selected invertebrate orders in one pair of plots. The general negative effect on ground beetles (but not beetles generally) that we detected might arise because they are prey species, whose numbers predicted pheasant chick survival (Hill & Robertson, 1988). They may also decrease due to damage to vegetation or nutrient changes in areas of high‐release density (Hall et al., 2021). The higher levels of day‐flying Lepidoptera may be explained by habitat management around release sites, with more butterflies found in areas of woodland managed for game than non‐game areas of the same woods (Robertson et al., 1988) or in game woods compared with non‐game woods (in some areas; Woodburn & Sage, 2005). Therefore, when examining any effects of released gamebirds on invertebrates, it would be critical to separate out taxa of interest and limit conclusions about effects to those specific groups.

Species of farmland and woodland birds used as indicators by DEFRA showed higher levels of total abundance, species richness, and Shannon diversity in RGS compared with CGS. This matches some effects on woodland specialist species from site‐specific studies showing (some) such species being more abundant in game‐managed woodland compared to nearby unmanaged areas (Draycott et al., 2008; Hoodless et al., 2006; Robertson, 1992; Robertson et al., 1988; Sage, 2018; Woodburn & Robertson, 1990). However, Davey (2008) found no relationships between bird abundance and pheasant densities in woodlands or between woods with and without pheasant releases. Positive effects on farmland bird species may be explained by the provision of supplementary food or enhanced winter habitats such as game crops or hedges, with more birds being found in game crops than control areas (Henderson et al., 2003; Parish & Sotherton, 2004, 2008; Sage et al., 2005). However, in hedges near high‐density release sites, there were fewer birds recorded, likely because of damage to hedge structure (Sage et al., 2009). The positive effects were seen when we considered absolute record data. For woodland birds, these effects were negative when we considered relative record data. This may be an artifact arising because the greater number of total bird records penalized any positive effects that existed. Conversely, if meaningful, it could be explained by exposure to disease carried by released birds (Gortázar et al., 2006) or competition for food due to dietary overlap (Bicknell et al., 2010) although these two mechanisms remain poorly understood.

Our study is necessarily crude for two reasons. First, the release data are almost certainly incomplete because it is likely that compliance with the Register is poor. The total number of birds being reported as held for release (~14.7 million) is around one‐third of the mean estimate of birds released calculated using a range of other methods (Madden, 2021). Therefore, the APHA release data are likely to underestimate the number of birds being released and, of more concern, likely to fail to record many locations where releases occur. While we can be confident that our RGS do contain released gamebirds, our CGS may be the sites of gamebird releases that have not been reported. We found that almost a fifth of CGS contained game crops. Some of these may be wildlife cropping, planted to attract or support farmland birds rather than game, but as we could not determine the crop composition of these strips we cannot be confident of each crops' purpose. Some of these may neighbor‐declared release sites, but others are likely the site of unregistered releases. This means that we might be less likely to detect effects of gamebird release because our set of Controls contains release sites. However, the APHA Poultry Register provides the only formal record of releases across the UK and therefore is currently the best dataset available for such analyses despite this confound. Clearly, there needs to be an improvement in the accuracy of registration to allow accurate assessments of ecological consequences of releases. Second, our biodiversity data was collected by a very large number of individuals and organizations, using different survey methods and with different original intentions. This is likely to introduce a range of skews and biases and citizen science datasets are acknowledged to be imperfect (e.g. Galván et al., 2021). However, this imprecision is offset by the national coverage afforded by the NBN Atlas and because the data were collected entirely blind to the hypotheses being tested by us. In a research area as publicly contentious as gamebird shooting, there is commonly a concern that data collection may be deliberately biased to support particular, preconceived views for or against shooting, either through the selection of study sites or choice of biodiversity sampling methods. We acknowledge that the data we use in our analyses is imperfect and urge that our results be treated with caution, especially regarding the magnitude of any effects which we detected.

How the differences in populations that we report are perceived and valued is likely to be subjective. For example, high numbers of rodents may be perceived as a source of agricultural pests and considered a negative consequence, or, at least in the UK, local population increases may be set against a general nationwide decline in small mammals (Coomber et al., 2021) and thus a positive consequence. Low fox numbers on RGS may be viewed as an undesirable perturbation of natural predator–prey relationships, or it may be used to refute the concern that gamebird release supports elevated populations of generalist predators and, given the threat that foxes can pose to some species of conservation concern, a positive consequence. A better appreciation of the net value of these effects may be possible if we understand the mechanisms by which release and management affect other species and we recommend setting the individual effects that we report within an ecological network that accounts for the direct, associated, and indirect effects of releasing and managing gamebirds (Madden & Sage, 2020). It may also help to contrast these outcomes with those arising from alternative land‐use options such as agriculture, forestry, or recreational land use.

We have found that, in Great Britain, the release and management of gamebirds can affect a wide range of non‐game species, driving decreases in some populations while increasing others, including some of conservation concern. These populations represent multiple trophic levels and include species occupying a range of lowland habitats including farmland and woodland. The practice of large‐scale gamebird release and management has persisted for at least a century in Great Britain, with a marked increase in scale over the past 50 years (Robertson et al., 2017), with similar patterns being seen in other countries. Consequently, it is likely to have had a persistent and pervasive effect on the populations of non‐game animals in areas where it occurs, contributing to the assemblages that we see today. Our work highlights the complex and holistic effects that release and management have across taxa. Both in the UK and worldwide, patterns and practices of gamebird release and management are likely to change markedly in the coming years, but the direction of those changes is unpredictable. One future scenario, if the recent trajectory continues (Robertson et al., 2017), is that more birds are released and those are managed more intensively. If so, game managers, game advisors, and legislators must be alert to likely negative effects for some non‐game populations and implement methods to ameliorate damage by developing, promoting, and following best practices relating to release sizes and densities, release site locations and the rearing condition of gamebirds. An alternative scenario is that, following public pressure, the release of gamebirds is restricted or banned, or the number and size of shoots decline as the supply of birds (often from Europe) is restricted by Brexit and/or avian flu. If so, those pressure groups calling for, or governing bodies implementing, such restrictions must acknowledge the risk that reduction or cessation of release and management may remove positive effects of, in particular, widescale habitat management, or introduce negative effects for some current populations. To mitigate these, they would need to propose viable ways to ensure the positive ecological effects are maintained despite the loss of motivation from gamebird release for land managers. In Great Britain, as in other countries where releases happen, our ignorance of the large‐scale effects, either positive or negative, is concerning, especially given the scale and history of this activity. This work serves to alert legislators, game managers, and campaigners to the diversity and complexity of the ecological consequences of this activity.

## AUTHOR CONTRIBUTIONS

**Joah Robert Madden:** Conceptualization (lead); formal analysis (equal); methodology (lead); project administration (lead); supervision (lead); visualization (equal); writing – original draft (lead); writing – review and editing (lead). **Rosie Buckley:** Data curation (equal); formal analysis (equal); visualization (equal); writing – original draft (supporting); writing – review and editing (supporting). **Sophia Ratcliffe:** Data curation (equal); writing – original draft (supporting); writing – review and editing (supporting).

## CONFLICT OF INTEREST STATEMENT

The APHAPR2020 dataset was obtained in conjunction with Dave Stone of Natural England. RB was funded by an internship from the Department of Psychology, University of Exeter. JRM conducted this research while supported by the Department of Psychology, University of Exeter. The NBN Atlas is funded by the National Biodiversity Network, with major contributions from Natural England, NatureScot, Northern Ireland Environment Agency, and Natural Resources Wales.

## Supporting information


Appendix S1

Appendix S2

Appendix S3

Appendix S4

Appendix S5

Appendix S6

## Data Availability

The location of release sites is protected due to privacy and security concerns. The data set was released by APHA to JRM under the condition of maintained anonymity, hence in the main data table “Appendix S5” the columns denoting site ID and coordinates have been blanked. The full data could be made available on request to JRM and with the explicit agreement of APHA.
