# Supplementary material for: Large‐scale correlations between gamebird release and management and animal biodiversity metrics in lowland Great Britain
Source: Ecol Evol. 2023 May 8;13(5):e10059. doi: 10.1002/ece3.10059 (PMC10166649; doi:10.1002/ece3.10059)
Supplement: Supplementary file 1 — Appendix S1 [file ECE3-13-e10059-s002.docx]

**Large-scale correlations between gamebird release and management and animal biodiversity metrics in lowland Great Britain**

Joah Robert Madden^1*^, Rosie Buckley^1^ and Sophia Ratcliffe^2^

**ESM Table of data providers of NBN Atlas records**

| Amphibian and Reptile Conservation | Freshwater Habitats Trust | Porcupine Marine Natural History Society |
| --- | --- | --- |
| Amphibian and Reptile Groups of the UK | Friends of the Earth | Pseudoscorpion Recording Scheme |
| Anthomyiid Recording Scheme | Froglife | Riverfly Recording Schemes |
| Aquatic Coleoptera Conservation Trust | Glasgow Museums Biological Records Centre | Royal Horticultural Society |
| Aquatic Heteroptera Recording Scheme | Gloucestershire Centre for Environmental Records | Royal Society for the Protection of Birds |
| Argyll Biological Records Centre | Grasshopper Recording Scheme | Salmon & Trout Conservation |
| Argyll Bird Club | Greenspace Information for Greater London CIC | Scotland's Environment Web |
| Balfour-Browne Club | Hebridean Whale and Dolphin Trust | Scottish Environment Protection Agency |
| Bat Conservation Trust | Hertfordshire Natural History Society Flora Group | Scottish Ornithologists' Club, The |
| Bedfordshire and Luton Biodiversity Recording and Monitoring¬†Centre | Highland Biological Recording Group | Scottish Wildlife Trust |
| Berkshire Moth Group | Hutchinson Ecological Associates | Seasearch |
| Biological Records Centre | Hypogean Crustacea Recording Scheme | Sheffield and Rotherham Wildlife Trust |
| BIS for Powys & Brecon Beacons National Park | Isle of Wight Local Records Centre | Shire Group of Internal Drainage Boards |
| Botanical Society of Britain & Ireland | JBA Consulting | Shropshire Ecological Data Network |
| Bristol Regional Environmental Records Centre | Joint Nature Conservation Committee | Silphidae Recording Scheme |
| British Bryological Society | Kent & Medway Biological Records Centre | Siphonaptera and Phthiraptera Recording Scheme |
| British Dragonfly Society Recording Scheme | Kent Wildlife Trust | Soldier Beetles and Allies Recording Scheme |
| British Lichen Society | Lancashire Environment Record Network | Soldierflies and Allies Recording Scheme |
| British Trust for Ornithology | Malcolm Storey | South East Wales Biodiversity Records Centre |
| Broadland Environmental Services Limited | Marine Biological Association | South West Scotland Environmental Information Centre (formerly DGERC) |
| Buglife | Marine Conservation Society | Steve Lane |
| Bumblebee Conservation Trust | Merseyside BioBank | Sussex Biodiversity Record Centre |
| Butterfly Conservation | Ministry of Justice | Tachinid Recording Scheme |
| Caledonian Conservation | National Plant Monitoring Scheme | Terrestrial Heteroptera Recording Scheme (Shieldbugs & allied species) |
| Cambridgeshire & Peterborough Environmental Records Centre | National Trust | Thames Valley Environmental Records Centre |
| Capturing our Coast | National Trust for Scotland | The Mammal Society |
| Central Scotland Green Network Trust | Natural Apptitude | The National Longhorn Beetle Recording Scheme |
| Centre for Environmental Data and Recording | Natural England | The Rock Pool Project |
| Chrysomelidae Recording Scheme | Natural Resources Wales | The Wildlife Information Centre |
| Cladocera Interest Group | NatureScot | UK Butterfly Monitoring Scheme |
| Cofnod ‚Äì North Wales Environmental Information Service | NatureSpot | UK Cranefly Recording Scheme |
| Conchological Society of Great Britain & Ireland | North East Scotland Biological Records Centre | University of Barcelona (Spain) |
| Cumbria Biodiversity Data Centre | Norwich Reptile Study Group | University of Reading |
| Derbyshire Biological Records Centre | Nottingham Urban Wildlife Scheme | Welsh Government |
| Dipterists Forum | Nottinghamshire Biological and Geological Records Centre | West Wales Biodiversity Information Centre |
| Dorset Environmental Records Centre | Oil Beetle Recording Scheme | Wild Surveys |
| Earthworm Society of Britain | OPAL | World Museum, National Museums Liverpool |
| Environment Agency | People's Trust for Endangered Species | Yorkshire Naturalists' Union |
| Freshwater Fish Recording Scheme | Plantlife | Yorkshire Wildlife Trust |
|  |  |  |
|  |  |  |
