# Supplementary material for: Large‐scale correlations between gamebird release and management and animal biodiversity metrics in lowland Great Britain
Source: Ecol Evol. 2023 May 8;13(5):e10059. doi: 10.1002/ece3.10059 (PMC10166649; doi:10.1002/ece3.10059)
Supplement: Supplementary file 2 — Appendix S2 [file ECE3-13-e10059-s004.docx]

**Large-scale correlations between gamebird release and management and animal biodiversity metrics in lowland Great Britain**

Joah Robert Madden^1*^, Rosie Buckley^1^ and Sophia Ratcliffe^2^

**ESM Data resources from the NBN Atlas.**

Amphibian and Reptile Conservation Trust, 2016.

ARG UK (2021). Record Pool. http://doi.org/10.15468/zen1gd

Allain, S. J. R. (2019). Mining Flickr: a method for expanding the known distribution of invasive species. Herpetological Bulletin, 148, 11-14 http://doi.org/10.15468/hrykhs

Anthomyiidae Recording Scheme (2021) records via iRecord. http://doi.org/10.15468/c5xmi2

Amphibian and Reptile Conservation Trust, 2016. http://doi.org/10.15468/uyjpfm

Amphibian and Reptile Conservation Trust, 2016. http://doi.org/10.15468/txftfx

Argyll Biological Records Centre (2021). Argyll Biological Records Dataset. http://doi.org/10.15468/ejve6c

Argyll Bird Club (2021). Bird records for Argyll for the years 2002 and 2003. http://doi.org/10.15468/uilybi

Balfour-Browne Club (2021). Water Beetle Surveys from Britain and Ireland. http://doi.org/10.15468/npcgrp

Bat Conservation Trust (2021). Waterway Survey. http://doi.org/10.15468/csxv5i

Bat Conservation Trust - National Bat Monitoring Programme http://doi.org/10.15468/39vba0

Natural England bat roost visit records from 2013 onwards, BCT (2019) http://doi.org/10.15468/oy8u34

National Bat Monitoring Programme (2021-06-18) Sunset/Sunrise Survey, Bat Conservation Trust. http://doi.org/10.15468/brmbw2

Bat Conservation Trust (2021). National Bat Monitoring Programme Roost Count. http://doi.org/10.15468/vm4psx

Bat Conservation Trust (2021). Field Survey. http://doi.org/10.15468/pmhdwn

Bat Conservation Trust (2021). Bechstein's Bat Survey Project. http://doi.org/10.15468/a6jgbl

Bat Conservation Trust - National Bat Monitoring Programme (2018) http://doi.org/10.15468/mizzy7

Bat Conservation Trust (2021). Woodland Survey. http://doi.org/10.15468/0q64zu

Bedfordshire and Luton Biodiversity Recording and Monitoring¬†Centre (2021). Bedfordshire Himalayan Balsam Surveys (WT) - 2010-2012. Occurrence dataset on the NBN Atlas

Berkshire Moth Group. 2021-06-18 Records verified via iRecord. http://doi.org/10.15468/mjrypq

Biological Records Centre. 2021-06-18. Bee, wasp and ant (Hymenoptera: Aculeata) records verified via iRecord. http://doi.org/10.15468/idirhy

Biological Records Centre. 2021-06-18. Tachinid Fly records verified via iRecord. http://doi.org/10.15468/qqw4va

Biological Records Centre (2021). UK Ladybird Survey data from iRecord. http://doi.org/10.15468/trfsfu

Biological Records Centre (2021). Grasshopper and Cricket (Orthoptera) and related species records from Britain and Ireland to 2007. http://doi.org/10.15468/nvyurg

National Trichoptera (Caddisfly) Recording Scheme (2021). Caddisfly (Trichoptera) records from Britain (excluding Northern Ireland and Channel Islands) up to the end of 2020 from the National Trichoptera (Caddisfly) Recording Scheme http://doi.org/10.15468/aqe7ua

Biological Records Centre. Diptera records from iRecord for families not covered by a recording scheme. http://doi.org/10.15468/iwy9yi

Ground Beetle Recording Scheme. 2021 Records verified via iRecord. http://doi.org/10.15468/gj8bkp

Biological Records Centre. 2021 Records shared via iRecord. http://doi.org/10.15468/yvgrpk

Biological Records Centre (2021). Cranefly (Diptera; Tipuloidea) records for Britain to 2016. http://doi.org/10.15468/wggm3t

Biological Records Centre (2021). Derek Lott Coleoptera Dataset. http://doi.org/10.15468/q13ilj

Biological Records Centre (2021). Carabidae records for Britain to 2014. http://doi.org/10.15468/5uuc0e

Biological Records Centre (2021). Millipede (Diplopoda) records for Britain and Ireland to 2005. http://doi.org/10.15468/27cr77

Biological Records Centre. 2021-06-18. Sawfly (Symphyta) records from iRecord. http://doi.org/10.15468/2v8r3b

Biological Records Centre (2021). Water Bug (aquatic Heteroptera) data for Britain. http://doi.org/10.15468/pffxpw

Biological Records Centre (2021). Caddisfly (Trichoptera) records from Britain and Ireland, via iRecord. http://doi.org/10.15468/llm4iq

Recording Invasive Species Counts (2021) RISC Heteroptera Non-Native Species Records. http://doi.org/10.15468/3tnk54

Biological Records Centre (2021). Opiliones (Harvestman) Dataset. http://doi.org/10.15468/ukhdhe

Biological Records Centre (2021). Pseudoscorpion Recording Scheme of the UK. http://doi.org/10.15468/kucfzl

Biological Records Centre (2021). Bob Merritt Coleoptera Dataset. http://doi.org/10.15468/hjlqbw

Recording Invasive Species Counts (2021) RISC Botanical Non-Native Species Records.

Biological Records Centre (2021). Cerambycidae Dataset. http://doi.org/10.15468/ghyhr7

Biological Records Centre. Coleoptera records from iRecord for families not covered by a recording scheme. http://doi.org/10.15468/qah8xl

Biological Records Centre (2021). Reptiles and Amphibians Dataset. http://doi.org/10.15468/1lhrhx

Biological Records Centre (2021). Database for the Atlas of Freshwater Fishes. http://doi.org/10.15468/3wfao2

Biological Records Centre (2021). Crayfish (Crustacea; Astacura) data for Britain and Ireland to 2003. http://doi.org/10.15468/aznmq1

Recording Invasive Species Counts (2021) RISC Non-Native Species Records for Muntjac. http://doi.org/10.15468/pcqzz8

Biological Records Centre (2021). HeathNet data. http://doi.org/10.15468/ikmfii

Rhinophoridae Recording Scheme. 2021 Records verified via iRecord. http://doi.org/10.15468/6gvpb2

Biological Records Centre (2021). Ticks (<i>Ixodidea</i>) distribution for the British Isles. Occurrence dataset on the NBN Atlas

Biological Records Centre (2021). Calliphoridae data from iRecord. Occurrence dataset on the NBN Atlas

Biological Records Centre (2021). Coccinellidae Data. http://doi.org/10.15468/6yuxsd

Recording Invasive Species Counts (2021) RISC Non-Native Species Records for Rhododendron Leafhopper. http://doi.org/10.15468/wrnn0v

BIS for Powys & Brecon Beacons National Park (2021). Brecknock county birds records. http://doi.org/10.15468/2rd8nh

Biodiversity Information Service for Powys and Brecon Beacons National Park (2021). INNS records held by BIS in Powys and Brecon Beacons National Park. Occurrence dataset accessed through the NBN Atlas. http://doi.org/10.15468/rhg0pc

Natural Resources Wales Regional Data : Mid-Wales, BIS (2020) http://doi.org/10.15468/whj6d7

BIS for Powys & Brecon Beacons National Park (2021). Powys Micro-moths. http://doi.org/10.15468/3sggqy

BIS for Powys & Brecon Beacons National Park (2021). Distribution of <i>Impatiens glandulifera</i> Royle along the river Irfon during June 2010. http://doi.org/10.15468/htab7e

Botanical Society of Britain and Ireland. 2021-06-18 Vascular plant records verified via iRecord. http://doi.org/10.15468/s4bje6

Botanical Society of Britain & Ireland (2021). Other BSBI Scottish data up to 2015. http://doi.org/10.15468/2dohar

Botanical Society of Britain & Ireland (2021). BSBI data from several English counties, up to 2015. http://doi.org/10.15468/yn7jkv

Botanical Society of Britain & Ireland (2021). Scottish SNH-funded BSBI records. http://doi.org/10.15468/llasrt

Bristol Regional Environmental Records Centre (2021). BEREC species records from all years at full resolution excluding Notable Species within the last 10 years. http://doi.org/10.15468/h1ln5p

Bristol Regional Environmental Records Centre (2021). BRERC Notable Species records within the last 10 years. http://doi.org/10.15468/vntgox

Blockeel TL, Bosanquet SDS, Hill M, Preston C (eds) 2014. Atlas of British and Irish bryophytes. Newbury: Pisces Publications. http://doi.org/10.15468/gvqhjb

Bryophyte data for Great Britain and Ireland from the British Bryological Society held by BRC: data compiled post-Atlas (2018) http://doi.org/10.15468/ttzehy

British Bryological Society Recording Scheme. 2021-06-18 Records verified via iRecord. http://doi.org/10.15468/2g3nyq

British Dragonfly Society Recording Scheme (2021). Dragonfly records from the British Dragonfly Society Recording Scheme. http://doi.org/10.15468/cuyjyi

British Lichen Society (2021). BLS Lichen Database: England 1650-2016. http://doi.org/10.15468/jskjlm

British Lichen Society (2021). BLS Rare and Threatened Lichen Records: England. http://doi.org/10.15468/pamwp1

British Lichen Society (2021). BLS Lichen Database: England churchyards 1650-2016. http://doi.org/10.15468/0duny2

British Lichen Society (2021). BLS Lichen Database: Wales 1650-2016. http://doi.org/10.15468/rbsztb

British Lichen Society (2021). BLS Lichen Database: Scotland 1700-2016. http://doi.org/10.15468/tbig5c

British Lichen Society (2021). BLS Rare and Threatened Lichen Records: Wales. http://doi.org/10.15468/eftzci

British Lichen Society (2021). BLS Lichen Database: Wales churchyards 1700-2016. http://doi.org/10.15468/mzl8ef

Records provided by BTO, accessed through NBN Atlas website

British Trust for Ornithology (2021). Biodiversity in Glasgow (BiG) Project. http://doi.org/10.15468/dju9iz

Broadland Environmental Services Limited (2021). Water vole distribution within Broadland (2003-2006): based upon field signs, sightings and trapping data. http://doi.org/10.15468/vjn3do

Broadland Environmental Services Limited (2021). Reptile distribution within Broadland (2004-2011): based upon field signs and sightings. http://doi.org/10.15468/y6zgrd

Broadland Environmental Services Limited (2021). Reptile distribution along the River Bure, Broadland (2010) . http://doi.org/10.15468/5yg0tg

Buglife (2021). Invertebrate records from sites that are mainly across Scotland. Occurrence record dataset on the NBN Atlas. http://doi.org/10.15468/aaxvmc

Bumblebee Conservation Trust (2020). BeeWalk bumblebee distribution records for Great Britain 2008-2019. Eastleigh, UK http://doi.org/10.15468/xde3qb

Butterfly distribution data from the Butterflies for the New Millennium recording scheme, courtesy of Butterfly Conservation and the Biological Records Centre. http://doi.org/10.15468/tqf8z3

Caledonian Conservation (2021). Caledonian Conservation Ltd Incidental Records 2014 onwards. http://doi.org/10.15468/1bigj0

Caledonian Conservation (2021). SNH Invertebrate Site Condition Monitoring 2015: Lurgie Loch SSSI. http://doi.org/10.15468/da57sa

Cambridgeshire & Peterborough Environmental Records Centre (2021). Invasive Non-Native Species. http://doi.org/10.15468/rg1l9v

Capturing our Coast (2021-06-18) Data verified via iRecord. http://doi.org/10.15468/zugq5h

Central Scotland Green Network Trust (2021). Invasive Non-native species data in the Clyde catchment, collated by Central Scotland Forest Trust. http://doi.org/10.15468/l6jkeo

Centre for Environmental Data and Recording (2021). CEDaR Online Recording. http://doi.org/10.15468/ke4kw8

Chrysomelidae Recording Scheme (2021-06-18) Chrysomelidae beetle data from iRecord. http://doi.org/10.15468/b9ghr2

Cladocera Interest Group (2021). Cladocera distribution updates 2014. http://doi.org/10.15468/czf3li

Cofnod - North Wales Environmental Information Service (2021). Records of Invasive Non Native Species held on the Cofnod database. http://doi.org/10.15468/nxdbhu

Cofnod - North Wales Environmental Information Service (2021). NRW Regional Data: North Wales. http://doi.org/10.15468/krljpu

Records captured from Licence Returns submitted to Natural Resources Wales (primarily mammal records), COFNOD (2019) http://doi.org/10.15468/turlas

Conchological Society of Great Britain & Ireland (2021), Conchological Society of Great Britain & Ireland: non-marine mollusc records. http://doi.org/10.15468/6dexp9

Conchological Society of Great Britain & Ireland (2021), Conchological Society of Great Britain & Ireland: marine mollusc records. Occurrence dataset http://doi.org/10.15468/aurwcz

Conchological Society of Great Britain & Ireland (2021), Conchological Society of Great Britain & Ireland: non-marine molluscs (fossil & subfossil records). http://doi.org/10.15468/b2ekhu

Cumbria Biodiversity Data Centre (2021). Cumbria Non Native Invasive Species. http://doi.org/10.15468/3exspu

Cumbria Biodiversity Data Centre (2021). Tullie House Museum Natural History Collections. http://doi.org/10.15468/epewfs

Derbyshire Biological Records Centre (2021). DBRC Hemiptera species records 1969 to 2015. http://doi.org/10.15468/gsxnep

Derbyshire Biological Records Centre (2021). Derbyshire Casual LEPIDOPTERA records - Casual records collated by Derby Museum.. http://doi.org/10.15468/6klknq

Derbyshire Biological Records Centre (2021). DBRC Hymenoptera species records 1975 to 2015. http://doi.org/10.15468/bkwgt8

Dipterists Forum (2021). Dipterists Forum - Field Weeks 2000 & 2001 (Cornwall & Devon). http://doi.org/10.15468/v5exhr

Dipterists Forum (2021). Dipterists Forum - Recording Scheme - Stilt & Stalk Flies. http://doi.org/10.15468/mwjnku

Dipterists Forum (2021). Sciomyzidae Recording Scheme. http://doi.org/10.15468/tx2mgd

Dipterists Forum (2021). Dipterists Forum: Field Week 2002 (Inverness). http://doi.org/10.15468/u6lxic

UK Sarcophagidae Recording Scheme. [Date] Records verified via iRecord. http://doi.org/10.15468/pbwpaa

Dipterists Forum (2021). Sepsidae Recording Scheme - records verified via iRecord. http://doi.org/10.15468/4rqjas

Heleomyzid Recording Scheme. [Date] Records verified via iRecord. http://doi.org/10.15468/yx9vbz

Sciomyzidae Recording Scheme http://doi.org/10.15468/5wegnv

Dipterists Forum (2021). Dipterists Forum: Field Week 2015 (Nottinghamshire). http://doi.org/10.15468/0d19t6

Dipterists Forum (2021). Dipterists Forum: Field Week 2016 (Canterbury). http://doi.org/10.15468/gcppmc

Hippoboscidae and Nycteribiidae Recording Scheme. 2021 Records verified via iRecord. http://doi.org/10.15468/ag3v64

Kelp Fly Recording Scheme. 2021 Records verified via iRecord. http://doi.org/10.15468/q2k74j

Sumner, D.P. 2020. Dipterists Forum Field Week 2018 Occurrence Dataset. http://doi.org/10.15468/2v9m9t

Dorset Environmental Records Centre (2021). Dorset SW Pilot species dataset. http://doi.org/10.15468/cpamwn

Dorset Environmental Records Centre (2021). Dorset Invasive Alien Plants - NBN South West Pilot Project Case Studies. http://doi.org/10.15468/8if4md

Forest Research (2020) Forest Research Earthworm Records (UK). Occurrence dataset http://doi.org/10.15468/zh7bvr

Earthworm Society of Britain (2020) Environment Agency Eiseniella tetraedra records (England). Occurrence dataset http://doi.org/10.15468/tsstqk

Earthworm Society of Britain (2020) National Earthworm Recording Scheme (UK). Occurrence dataset http://doi.org/10.15468/vss5gb

Earthworm Society of Britain (2020) Earthworm Research Group (UCLan) Earthworm Records (UK). Occurrence dataset http://doi.org/10.15468/pbw2eu

Earthworm Society of Britain (2021) Earthworm Research Records (UK). http://doi.org/10.15468/domlgi

Environment Agency (2021). Protected and Invasive Species Records Collected Through Environment Agency Survey 1995 - 2021. http://doi.org/10.15468/awfvnp

Environment Agency (2021). Environment Agency England and Wales (2007) freshwater invertebrate species (single species families). http://doi.org/10.15468/bmg5ex

Environment Agency (2021). England Non Native Species records 1965 to 2017. http://doi.org/10.15468/pdop0k

Environment Agency (2021). Crayfish data update for Environment Agency Thames Region March 2009. http://doi.org/10.15468/eambll

Freshwater Fish Recording Scheme (2021). Freshwater fish records via iRecord. http://doi.org/10.15468/eeafla

Freshwater Fish Recording Scheme (2021). Freshwater fish records via iRecord - additional species. http://doi.org/10.15468/pyjbo9

Freshwater Habitats Trust (2021). PondNet data 2012-2014. http://doi.org/10.15468/qv8ped

Friends of the Earth (2021). Great British Bee Count 2016 - Stage 2 (records verified from photo). http://doi.org/10.15468/gyvrzv

Friends of the Earth (2021). Great British Bee Count 2015 - Stage 2 (records verified from photo). http://doi.org/10.15468/c2c6st

Froglife (2021). Froglife's amphibian and reptile records from the Dragon Finder App for Great Britain in 2016-2020 (inclusive). http://doi.org/10.15468/niyja0

Glasgow Museums BRC: Mammals (2019). http://doi.org/10.15468/fphygc

Glasgow Museums BRC: Ladybirds (2019). http://doi.org/10.15468/95qyds

Glasgow Museums BRC: Chrysomelidae. (2019) http://doi.org/10.15468/9z4fv6

Glasgow Museums BRC: Hemiptera (2019) http://doi.org/10.15468/gvvxvz

GCER (2021). 1930-2013 Spider Records, Gloucestershire, England. Digitised from the handwritten card archive of the late County Spider Recorder David John Russell Haigh, with both expert and financial support from Gloucestershire Naturalists' Society. http://doi.org/10.15468/s8xsr4

Orthoptera Recording Scheme (2021) Orthoptera and Allied Insects of the British Isles data from iRecord. http://doi.org/10.15468/lyafx9

Greenspace Information for Greater London CIC (2018). http://www.gigl.org.uk/ http://doi.org/10.15468/kzxbmb

Hebridean Whale and Dolphin Trust (2021). Visual sightings data set 2003-2020. Occurrence dataset on the NBN Atlas

Hertfordshire Natural History Society Flora Group (2021). Hertfordshire Flora Survey Records 1987-2005. http://doi.org/10.15468/ntvxgr

Highland Biological Recording Group (2021). HBRG Insects Dataset. Occurrence dataset accessed through the NBN Atlas. http://doi.org/10.15468/u1p4wc

Highland Biological Recording Group (2021). HBRG Fungus, Lichen & Lower Plants Dataset. Occurrence dataset accessed through the NBN Atlas. http://doi.org/10.15468/rt48my

Highland Biological Recording Group (2021). Vertebrates (not Badger) Dataset. http://doi.org/10.15468/vaassa

Highland Biological Recording Group (2021). HBRG Other Invertebrates Dataset. http://doi.org/10.15468/r780rb

Highland Biological Recording Group (2021). HBRG Badger Dataset. http://doi.org/10.15468/llj6qk

Highland Biological Recording Group (2021). HBRG P.F. Entwistle Colln and Notebooks Dataset http://doi.org/10.15468/fmcwyl

Highland Biological Recording Group (2021). HBRG JP Blunt Fungus Dataset. http://doi.org/10.15468/w949ss

Hutchinson Ecological Associates (2021). heacology bat records. Occurrence dataset on the NBN Atlas

Hypogean Crustacea Recording Scheme (2021). Hypogean macro-Crustacea records. http://doi.org/10.15468/jzjzcr

Isle of Wight Local Records Centre (2021). Isle of Wight non-native invasive species. http://doi.org/10.15468/laa1k8

JBA Consulting (2021). ARPW schemes in Great Britain for the period 2014-2018. Occurrence dataset on the NBN Atlas

Joint Nature Conservation Committee (2021). England Otter Survey Database. http://doi.org/10.15468/9ij1ku

JNCC (2021). River Macrophytes Database. http://doi.org/10.15468/mebiar

Joint Nature Conservation Committee (2021). Wales Otter Survey Database. http://doi.org/10.15468/kl4tuv

Kent & Medway Biological Records Centre (2018). Coleoptera: Records for Kent. Occurrence dataset¬† http://doi.org/10.15468/qwl87e

Kent & Medway Biological Records Centre (2021). Orthoptera & Allies: Records for Kent. http://doi.org/10.15468/0rtrrm

Kent & Medway Biological Records Centre (2021). Fish: Records for Kent.. http://doi.org/10.15468/kd1utk

Kent Wildlife Trust (2021). Kent Wildlife Trust Shoresearch Intertidal Survey 2004 onwards. http://doi.org/10.15468/zyxxue

Lancashire Environment Record Network (2021). LERN Invasive Non-native Species Records. http://doi.org/10.15468/nt52o2

Malcolm Storey (2021). http://www.bioimages.org.uk/ Malcolm Storey personal records and images. Occurrence dataset on the NBN Atlas

Verified marine records from Indicia-based surveys by Marine Biological Association and Biological Records Centre under licence CC BY. Released under DASSH terms and conditions. See http://www.dassh.ac.uk/terms-and-conditions http://doi.org/10.15468/yfyeyg

DASSH Data Archive Centre Academic Surveys by Marine Biological Association under licence CC-BY

Released under DASSH terms and conditions. See http://www.dassh.ac.uk/terms-and-conditions http://doi.org/10.15468/cwqszy

RISC and ALERT Marine Non-Native Species (Chinese Mitten Crab, Wakame and Carpet Sea Squirt) Records by Marine Biological Association under CC-BY

Released under DASSH terms and conditions. See http://www.dassh.ac.uk/terms-and-conditions http://doi.org/10.15468/ls37zx

2005 - Ongoing United Kingdom MarLIN Shore Thing timed search results by Marine Biological Association under licence CC BY.

Released under DASSH terms and conditions. See http://www.dassh.ac.uk/terms-and-conditions http://doi.org/10.15468/ikwlzr

DASSH Data Archive Centre expert sightings records by Marine Biological Association under licence CC-BY

Released under DASSH terms and conditions ‚Äì See http://www.dassh.ac.uk/terms-and-conditions http://doi.org/10.15468/tggq3w

DASSH Data Archive Centre volunteer sightings records by Marine Biological Association under licence CC BY.

Released under DASSH terms and conditions ‚Äì See http://www.dassh.ac.uk/terms-and-conditions http://doi.org/10.15468/xwiw3h

Bishop, JDD, Wood, CA, Yunnie, ALE, Griffiths, CA. (2015) Unheralded arrivals: non-native sessile invertebrates in marinas on the English coast. Aquatic Invasions, 10. http://doi.org/10.15468/t9edux

Marine Conservation Society (2021). UK Basking Shark sightings from 1987 to 2016. Occurrence dataset on the NBN Atlas

Marine Conservation Society (2021). UK Jellyfish Sightings from 2003 to 2015. Occurrence dataset on the NBN Atlas

Merseyside BioBank (2021). North Merseyside Local Species Data (DEFRA Grant 23169). http://doi.org/10.15468/h5k3ix

Ministry of Justice (2021). Species found in the NOMS estate 2005 - Present. http://doi.org/10.15468/wcx4is

National Plant Monitoring Scheme (2021). National Plant Monitoring Scheme - England, Scotland, Wales. http://doi.org/10.15468/jz6s6t

National Trust (2021). National Trust Species Records. http://doi.org/10.15468/opc6g1

National Trust for Scotland (2021). National Trust for Scotland Species Records. http://doi.org/10.15468/a5y1cz

Natural Apptitude (2021). PlantTracker data from 2012 onwards. http://doi.org/10.15468/8y4bie

Natural Apptitude (2021). AquaInvaders. http://doi.org/10.15468/nagz1b

Natural Apptitude (2021). Sealife Tracker. http://doi.org/10.15468/qgk3pg

Natural England (2021). Invertebrate Common Standards Monitoring and ISIS Test Data. http://doi.org/10.15468/epw9ym

Natural England (2021). Riperia. Natural England copyright. Contains Ordnance Survey data Crown copyright and database right 2021 http://doi.org/10.15468/wunv9t

Natural England (2021). Bat sites inventory for England (1949-2011). http://doi.org/10.15468/glmmky

Natural England (2021). Environmental Monitoring Database Species data 1987 to 2015. http://doi.org/10.15468/xu5bmy

Natural England iRecord Surveys (2021) http://doi.org/10.15468/i7x5ca

Natural England (2018). Great crested newt presence records determined from Class Licence return documents. Accessed on 2021-06-18 at https://registry.nbnatlas.org/public/show/dr1939. http://doi.org/10.15468/xe8xni

Natural England (2021) Invertebrate Site Register - England (1738-2005). http://doi.org/10.15468/7wbiu7

Natural England (2021). Natural England Marine Monitoring surveys. http://doi.org/10.15468/ysikg5

Natural England Long-Term Monitoring Network Vegetation Quadrats (2019) http://doi.org/10.15468/eiazkc

Natural England (2021). Norfolk and Suffolk Broads Invertebrate Data 2007-2008. http://doi.org/10.15468/uloykk

Natural Resources Wales. (2021) Natural Resources Wales (NRW) Licence Return Dataset. Occurrence dataset accessed through the NBN Atlas. http://doi.org/10.15468/wlg2vm

Natural Resources Wales. (2021) Phase 2 Lowland Grassland Survey of Wales. Occurrence dataset accessed through the NBN Atlas. http://doi.org/10.15468/segig2

Natural Resources Wales. (2021) Rare and Protected Species Records Across Wales 1975 to 2012. Occurrence dataset accessed through the NBN Atlas. http://doi.org/10.15468/q2fbns

Natural Resources Wales. (2021) KiEco Freshwater Ecology: River Macrophytes. Occurrence dataset accessed through the NBN Atlas. http://doi.org/10.15468/h5fjud

Natural Resources Wales. (2021) KiEco Freshwater Ecology: River Macroinvertebrates. Occurrence dataset accessed through the NBN Atlas. http://doi.org/10.15468/qggn2f

Natural Resources Wales. (2021) Native Species Surveys Across Wales (1978 to 2006). Occurrence dataset accessed through the NBN Atlas. http://doi.org/10.15468/nzfctb

Natural Resources Wales. (2021) Welsh Invertebrate Database (WID). Occurrence dataset accessed through the NBN Atlas. http://doi.org/10.15468/bv8fcj

Natural Resources Wales. (2021) Ad-hoc Bat Records. Occurrence dataset accessed through the NBN Atlas. http://doi.org/10.15468/q1ob6g

Natural Resources Wales. (2021) KiEco Freshwater Ecology: Diatoms. Occurrence dataset accessed through the NBN Atlas. http://doi.org/10.15468/i5w6np

Natural Resources Wales. (2021) UK Biodiversity Action Plan Invertebrate Data for Wales. Occurrence dataset accessed through the NBN Atlas. http://doi.org/10.15468/3qrrks

Natural Resources Wales. (2021) Marine data from Natural Resources Wales (NRW) Technical Support (Research & Monitoring) Contracts, Wales. Occurrence dataset accessed through the NBN Atlas. http://doi.org/10.15468/az7nw3

Natural Resources Wales. (2021) Marine Intertidal Phase 1 Species Dataset from the Countryside Council for Wales 1996-2005. Occurrence dataset accessed through the NBN Atlas. http://doi.org/10.15468/kflo7m

Natural Resources Wales. (2021) Phase 2 Lowland Peatland Survey of Wales 2004 onwards. Occurrence dataset accessed through the NBN Atlas. http://doi.org/10.15468/ijegqu

Natural Resources Wales. (2021) Freshwater Lamprey Survey Data. Occurrence dataset accessed through the NBN Atlas. http://doi.org/10.15468/zsv2ob

Natural Resources Wales. Freshwater Bullhead Survey Data in Wales. (2021). Occurrence dataset accessed through the NBN Atlas. http://doi.org/10.15468/dgwfke

Natural Resources Wales. (2021) Non Native Species Across Wales (1989 to 2012). Occurrence dataset accessed through the NBN Atlas. http://doi.org/10.15468/ekppsa

Natural Resources Wales. (2021) Freshwater Shad Monitoring Data. Occurrence dataset accessed through the NBN Atlas. http://doi.org/10.15468/owttzo

NatureScot (2021). Wild boar sightings held by Andy Riches. http://doi.org/10.15468/sxwhjm

SNH (2008). National Water Vole Database & Mapping Project (Scotland data) http://doi.org/10.15468/dm7vff

SNH (2020). Bat Casework records 1970-2007 http://doi.org/10.15468/ogugxm

NatureScot (2021). New Zealand Flatworm (Arthurdendyus triangulatus) records for Scotland 2008-2014. http://doi.org/10.15468/7rlf4q

Reynolds, P. & Harris, M. (2005). Inverness Badger Survey 2003. Scottish Natural Heritage Commissioned Report No. 096 http://doi.org/10.15468/wkpu94

NatureScot (2021). SNH Bat Casework Recording log 2015. http://doi.org/10.15468/lkivcj

NatureScot (2021). Survey of distribution of beavers in Tayside in 2012. http://doi.org/10.15468/7hmxga

Campbell-Palmer, R., Puttock, A., Graham, H., Wilson, K., Schwab, G., Gaywood, M.J. & Brazier, R.E. (2018). Survey of the Tayside area beaver population 2017-2018. Scottish Natural Heritage Commissioned Report No. 1013. www.nature.scot/snh-research-report-1013-survey-tayside-area-beaver-population-2017-2018 http://doi.org/10.15468/qt4eej

NatureScot (2021). Reports of New Zealand Flatworms in Scotland, 1989 - 2005. http://doi.org/10.15468/6gx3pq

NatureScot (2021). Expansion zone survey of pine marten (Martes martes) distribution in Scotland, 2012-2013. http://doi.org/10.15468/rpymvd

Proctor, K. & Rafferty, T.F. (2004). Survey of Giant Hogweed, Japanese Knotweed and Himalayan Balsam on five major Lothian rivers. Scottish Natural Heritage Commissioned Report No. 223 http://doi.org/10.15468/5ypgap

NatureScot (2021). Records of otters from Site Condition Monitoring in Scotland 2011-2012. http://doi.org/10.15468/3kuoll

NatureScot (2020). Species data for Scottish waters held and managed by Scottish Natural Heritage, derived from benthic surveys 1993 to 2018 http://doi.org/10.15468/faxvgd

NatureScot (2021). SNH Bat Casework Recording log 2016. http://doi.org/10.15468/jcw23j

Lavery, L. (2016) Juniper survey of Perth and Kinross, 2010. Scottish Natural Heritage Commissioned Report No. 920 http://doi.org/10.15468/exhdod

NatureScot (2021). Wild boar SASA sightings record. http://doi.org/10.15468/puvwej

NatureScot (2021). SNH Bat Casework Recording log 2014. http://doi.org/10.15468/fhgfxu

NatureScot (2021). Site Condition Monitoring Records from surveys of lamprey species in five river SACs in Scotland. http://doi.org/10.15468/70rz8i

Carss, D.N./Centre for Ecology and Hydrology (2004) Survey and identification of sites of importance to otter in the catchment of the River Dee. Scottish Natural Heritage Commissioned Report F03L08. http://doi.org/10.15468/ekeqbp

NatureScot (2021). Stoats in Orkney Facebook page records & sightings, 2014 - 2015. http://doi.org/10.15468/kwivrh

National Lamprey Survey of Scotland, SNH (2020) http://doi.org/10.15468/gbeajh

NatureSpot (2021). All taxa records for Leicestershire and Rutland. http://doi.org/10.15468/i46are

North East Scotland Biological Records Centre (2021). North East Scotland Birds (1883-2010). http://doi.org/10.15468/o1okcz

North East Scotland Biological Records Centre (2021). North East Scotland Terrestrial Mammals 1900-2017 (excluding squirrels, wild cats and marine mammals). http://doi.org/10.15468/qt0mly

North East Scotland Biological Records Centre (2021). NE Scotland butterfly and moth records 1800-2010. http://doi.org/10.15468/7nnp7d

North East Scotland Biological Records Centre (2021). North East Scotland Invasive Non Native Plants. http://doi.org/10.15468/oopmk2

North East Scotland Biological Records Centre (2021). North East Scotland Mosses and Liverworts (1950-2014). http://doi.org/10.15468/wgzruc

North East Scotland Biological Records Centre (2021). Dragonflies and Damselflies 1862-2013. http://doi.org/10.15468/aj5vv7

North East Scotland Biological Records Centre (2021). North East Scotland Amphibians & Reptiles 1850 - 2016. http://doi.org/10.15468/ruf7hc

North East Scotland Biological Records Centre (2021). NE Scotland other invertebrate records 1800-2010. http://doi.org/10.15468/ifjfxz

North East Scotland Biological Records Centre (2021). NE Scotland fungus and lichen records 1800-2010. http://doi.org/10.15468/v6mt0g

North East Scotland Biological Records Centre (2021). NE Scotland fish records 1800-2010. http://doi.org/10.15468/kjrwnd

Norwich Reptile Study Group (2021). Norwich Reptile Study Group Records for 2011. http://doi.org/10.15468/ajovn4

Norwich Reptile Study Group (2021). Norwich Reptile Study Group Records for 2010. http://doi.org/10.15468/wdqjin

Nottingham Urban Wildlife Scheme iRecord download 2014 - 2017 (2019) http://doi.org/10.15468/sblbwa

Nottinghamshire Biological and Geological Records Centre (2021). Nottinghamshire Non-Native Invasive Species Dataset. Occurrence dataset on the NBN Atlas

Nottinghamshire Biological and Geological Records Centre (2021). Bob Merritt dataset of Nottinghamshire invertebrates. http://doi.org/10.15468/1pcd8f

Nottinghamshire Biological and Geological Records Centre (2021). Nottinghamshire Biodiversity Action Group Brown Hare Survey. Occurrence dataset on the NBN Atlas

Nottinghamshire Biological and Geological Records Centre (2021). Trevor and Dilys Pendleton Eakring and Nottinghamshire Invertebrate Records. Occurrence dataset on the NBN Atlas

Oil Beetle Recording Scheme. 2021 Records verified via iRecord. http://doi.org/10.15468/r844yt

OPAL (2021). OPAL New Zealand Flatworm Survey - flatworm sightings. http://doi.org/10.15468/p2nvb2

People's Trust for Endangered Species (2021). Big Hedgehog Map records. http://doi.org/10.15468/omvfyk

People's Trust for Endangered Species (2021). Hedgehog records 2005-2006: Hogwatch and Rural Landowner surveys. http://doi.org/10.15468/67xdvh

People's Trust for Endangered Species Living with Mammals survey (2019) http://doi.org/10.15468/kv7kgm

People's Trust for Endangered Species (2021). National Dormouse Database (NDD). http://doi.org/10.15468/kcty0c

People's Trust for Endangered Species (2020). Other Mammal Data from National Dormouse Monitoring Programme (NDMP). http://doi.org/10.15468/v5uvuc

People's Trust for Endangered Species (2021). Hedgehog Hibernation Survey records. http://doi.org/10.15468/6ncy5o

People's Trust for Endangered Species (2021). National Water Vole Monitoring Programme (NWVMP). http://doi.org/10.15468/l5l7yp

People's Trust for Endangered Species Other beetle records from National Stag Beetle Surveys 2006-2019 (2020) http://doi.org/10.15468/fsie6a

People's Trust for Engangered Species (2021). National Stag Beetle Surveys (Great Stag Hunts). http://doi.org/10.15468/ovzeen

People's Trust for Endangered Species (2018). PTES Noble Chafer: Post-BAP records from 1998 onwards. http://doi.org/10.15468/hw1bxa

People's Trust for Endangered Species (2021). PTES - incidental Coleoptera records from the 2006-2007 National Stag Beetle Survey. http://doi.org/10.15468/apdozd

Plantlife (2021). Back from the Brink vascular plant species abundance and distribution for Great Britain for the period 2002-2009. http://doi.org/10.15468/xmgwgo

Porcupine Marine Natural History Society (2020): Porcupine Marine Natural History Society Dataset. v2.2. Marine Biological Association. Dataset/Samplingevent. https://doi.org/10.17031/zay9rc http://doi.org/10.15468/pcmg9q

Pseudoscorpion Recording Scheme 2021. Records verified via iRecord. http://doi.org/10.15468/mggbb2

Riverfly Recording Schemes (2021). Mayfly (Ephemeroptera) Data for Great Britain for 1906-2017. http://doi.org/10.15468/wmigy8

Riverfly Recording Schemes (2021). Mayfly (Ephemeroptera) records from Britain and Ireland, via iRecord. http://doi.org/10.15468/bdmxpk

Riverfly Recording Schemes (2021). Stonefly (Plecoptera) records from Britain and Ireland, via iRecord. http://doi.org/10.15468/ptw1tt

iNaturalist & UK Trichoptera (Caddisfly) Recording Scheme (2021). Caddisfly records from iNaturalist verified by the UK Trichoptera (Caddisfly) Recording Scheme. Occurrence dataset on the NBN Atlas

Royal Horticultural Society (2021). RHS lily beetle (Lilioceris lilii) monitoring. http://doi.org/10.15468/vxglmj

Royal Horticultural Society (2021). RHS Fuchsia Gall Mite (Aculops fuchsiae) monitoring. http://doi.org/10.15468/25sssx

Royal Horticultural Society (2021). RHS berberis sawfly (Arge berberidis) monitoring. http://doi.org/10.15468/rxjz7m

Royal Horticultural Society (2021). RHS Hemerocallis midge survey. http://doi.org/10.15468/gt2j0y

Royal Horticultural Society (2021). RHS rosemary beetle (Chrysolina americana) monitoring. http://doi.org/10.15468/spnw1a

Royal Horticultural Society (2021). European Pear Rust (<i>Gymnosporangium sabinae</i>) monitoring. http://doi.org/10.15468/hsyzkm

Royal Horticultural Society (2021). RHS Box tree moth (Cydalima perspectalis) monitoring. http://doi.org/10.15468/wg9l2v

Royal Horticultural Society http://doi.org/10.15468/b7sk69

RSPB (2021). 1921 Onwards Swift Screaming Parties, UK. http://doi.org/10.15468/peedv8

RSPB (2021). 1921 Onwards Occupied Swift Nests, UK. http://doi.org/10.15468/3es6ec

RSPB (2021). 1989 Onwards Previously Occupied Swift Nests, UK. Occurrence dataset on the NBN Atlas

RSPB (2021). 2015 onwards Swift Nest Boxes, UK. Occurrence dataset on the NBN Atlas

Royal Society for the Protection of Birds (2021). Farmland bird database in Dumfries and Galloway, 1999-2011. http://doi.org/10.15468/wrwwke

Royal Society for the Protection of Birds (2021). Tree sparrow recovery project summary data, North West England, 2004-2010. http://doi.org/10.15468/l1memj

Royal Society for the Protection of Birds (2021). Farmland Bird Survey North East Wales, 2008. http://doi.org/10.15468/ktjmql

Please acknowledge RSPB and local volunteer groups for each region in any use of the data. Please see the survey level metadata for further details of individuals and groups involved. http://doi.org/10.15468/4z5epz

RPSB (2021). Corncrake national surveys - raw data. http://doi.org/10.15468/oqwqca

Data reproduced with the permission of RSPB http://doi.org/10.15468/iflvjc

Please acknowledge the Royal Society for the Protection of Birds (RSPB) and Natural England in the use of this data. http://doi.org/10.15468/qjx9zc

RSPB (2021). Corncrake national surveys - territory centres. http://doi.org/10.15468/wgcc49

Data reproduced with the permission of RSPB http://doi.org/10.15468/ssn1l9

Please acknowledge the Royal Society for the Protection of Birds (RSPB), Scottish Natural Heritage (SNH) and SCARABBS in any use of the data. http://doi.org/10.15468/rvh2gx

Royal Society for the Protection of Birds (2021). Turtle Dove sightings from research project in East Anglia, 2010. http://doi.org/10.15468/ssujxr

Salmon & Trout Conservation. (2019). Riverfly Census [Dataset] http://doi.org/10.15468/dz1qvj

Scotland's Environment Web (2021). Invasive non-native species records from SEWeb. Occurrence dataset on the NBN Atlas

Scottish Environment Protection Agency 2015, licensed under the Open Government Licence v3.0 http://doi.org/10.15468/l82tvb

Scottish Environment Protection Agency 2015, licensed under the Open Government Licence v3.0 http://doi.org/10.15468/knxcqi

Scottish Environment Protection Agency 2015, licensed under the Open Government Licence v3.0 http://doi.org/10.15468/jb73hj

Scottish Ornithologists' Club, The (2021). Highland Breeding Bird Data 2002-2003 - non-sensitive breeding species. http://doi.org/10.15468/559t1y

Scottish Wildlife Trust (2021). The Scottish Squirrel Database. Occurrence dataset accessed through the NBN Atlas. http://doi.org/10.15468/fqg0h3

Scottish Wildlife Trust (2021). Commissioned surveys and staff surveys and reports for Scottish Wildlife Trust reserves - Verified data. http://doi.org/10.15468/a6snhl

Commissioned surveys and staff surveys and reports for Scottish Wildlife Trust reserves - Unassessed data, SWT (2020) http://doi.org/10.15468/dfwjgc

Scottish Wildlife Trust (2021). Survey and monitoring records for Scottish Wildlife Trust reserves from reserve convenors and Trust volunteers - Verified data. http://doi.org/10.15468/yyd4b9

Scottish Wildlife Trust (2021). Survey and monitoring records for Scottish Wildlife Trust reserves from reserve convenors and Trust volunteers - Unassessed data. http://doi.org/10.15468/jxrydj

Seasearch (2021). Seasearch Marine Surveys in England. http://doi.org/10.15468/kywx6m

Seasearch (2021). Seasearch Marine Surveys in Wales. http://doi.org/10.15468/4us2hk

Seasearch (2021). Seasearch Marine Surveys in Scotland. http://doi.org/10.15468/0hyjxi

Data supplied by Sheffield and Rotherham Wildlife Trust http://doi.org/10.15468/x3wdpp

Shire Group of Internal Drainage Boards (2021). Shire Group IDB species data 2004 to present. http://doi.org/10.15468/p3ismw

Shropshire Ecological Data Network (2021). Shropshire Ecological Data Network database. http://doi.org/10.15468/5v5pvk

Silphidae Recording Scheme (2021). Silphidae Recording Scheme - data verified via iRecord. http://doi.org/10.15468/h6nbqe

Siphonaptera and Phthiraptera Recording Scheme. 2021 Records verified via iRecord. http://doi.org/10.15468/sa4824

Soldier Beetles and Allies Recording Scheme 2021. Records verified via iRecord. http://doi.org/10.15468/7ms3bt

Soldierflies and Allies Recording Scheme ([data of download]). Records via iRecord. http://doi.org/10.15468/6pghte

SEWBReC (2020). INNS Data: All Taxa (South East Wales). http://doi.org/10.15468/crhjs2

SEWBReC (2021). NRW Regional Data: South East Wales Non-sensitive species. http://doi.org/10.15468/g7xxs8

SWSEIC (2020). Bob Merritt invertebrate records for VCs 72-75 http://doi.org/10.15468/a35rnz

Steve Lane - personal records and dataset for Warwickshire Coleoptera and Hemiptera (2020) http://doi.org/10.15468/jhgun5

Sussex Biodiversity Record Centre (2020). An 'as-complete-as-possible' Sussex Beetle Records dataset covering the period to March 2017. http://doi.org/10.15468/nabhzq

Sussex Biodiversity Record Centre (2021). Patrick Roper‚Äôs Notebooks. http://doi.org/10.15468/ntnedq

Tachinid Recording Scheme (2021). Tachinid Recording Scheme - GB and Ireland records. http://doi.org/10.15468/xps6uq

Terrestrial Heteroptera Recording Scheme (2021). Shieldbug & allied species records, from iRecord. http://doi.org/10.15468/axiomi

Terrestrial Heteroptera Recording Scheme (2020). Flickr (01) Shieldbugs & allied species. http://doi.org/10.15468/xmfn5j

Terrestrial Heteroptera Recording Scheme (Shieldbugs & allied species) (2021). iSpot (2008-2010): Shieldbugs & allied species. http://doi.org/10.15468/fdq9rl

Thames Valley Environmental Records Centre (2020). TVERC Historical Churchyard Records

National Mammal Atlas Project, online recording (2021) http://doi.org/10.15468/i2eosa

Mammal Society 2021 Mammal Mapper App Sighting Records http://doi.org/10.15468/mjw32e

The National Longhorn Beetle Recording Scheme (2021). Longhorn beetle (Cerambycidae) data from iRecord. http://doi.org/10.15468/p8f9ww

The Rock Pool Project database - www.therockpoolproject.co.uk - downloaded 2021-06-18 http://doi.org/10.15468/bqpd1n

The Wildlife Information Centre (2019). TWIC Biodiversity Field Trip Data (1995-present). http://doi.org/10.15468/ljc0ke

The Wildlife Information Centre (2020). TWIC Site Surveys (2010 - present)

The Wildlife Information Centre (2020). TWIC Hedgehog Public Survey. http://doi.org/10.15468/p2aosu

The Wildlife Information Centre (2020). TWIC Spots and Stripes Public Survey.

The Wildlife Information Centre (2020). TWIC Record a Raptor Survey. http://doi.org/10.15468/drsfui

The Wildlife Information Centre (2019). R. Manning's Records

The Wildlife Information Centre (2019). TWIC - Identification Workshops dataset. http://doi.org/10.15468/pplu90

The Wildlife Information Centre (2021). Ron McBeath records (2010 - 2014). Occurrence dataset on the NBN Atlas

The Wildlife Information Centre (2020). TWIC General Records (2015 - present)

The Wildlife Information Centre (2021). City of Edinburgh Natural Heritage Service - Natural Heritage Service Data. http://doi.org/10.15468/lexela

The Wildlife Information Centre (2021). City of Edinburgh Natural Heritage Service - Ranger Patrol Records. http://doi.org/10.15468/j9ht0u

The Wildlife Information Centre (2021). City of Edinburgh Natural Heritage Service - General Public Records.. http://doi.org/10.15468/mfkmtz

The Wildlife Information Centre (2020). East Lothian Council - Woodland Plants Survey 2011 (public)

The Wildlife Information Centre (2020). East Lothian Council Ranger Service Records

The Wildlife Information Centre (2020). East Lothian Council - Biodiversity Surveys

The Wildlife Information Centre or BRISC (depends on orginator in dataset name) http://doi.org/10.15468/t50yhv

Contains UK Butterfly Monitoring Scheme (UKBMS) data © copyright and database right Butterfly Conservation, the Centre for Ecology & Hydrology, British Trust for Ornithology, and the Joint Nature Conservation Committee http://doi.org/10.15468/jgm7ci

Contains UK Butterfly Monitoring Scheme (UKBMS) data © copyright and database right Butterfly Conservation, the Centre for Ecology & Hydrology, British Trust for Ornithology, and the Joint Nature Conservation Committee. http://doi.org/10.15468/gmqvmk

UK Cranefly Recording Scheme 2021. Records verified via iRecord. http://doi.org/10.15468/mjsdup

University of Barcelona (Spain) (2021). Charipinae from UK. http://doi.org/10.15468/hsuivu

University of Reading (2021). Powdery Mildew Citizen Science Survey, UK, 2013-2015. http://doi.org/10.15468/8eao8z

Welsh Government (2021). Tir Cynnal and Tir Gofal Monitoring and Evaluation Programme: Field Survey; farm-scale survey for noctule, common pipistrelle and soprano pipistrelle. Occurrence dataset on the NBN Atlas

Welsh Government (2021). Tir Cynnal and Tir Gofal Monitoring and Evaluation Programme: Arable plant survey. Occurrence dataset on the NBN Atlas

Welsh Government (2021). Tir Cynnal and Tir Gofal Monitoring and Evaluation Programme: Field-scale surveys for breeding yellowhammer. Occurrence dataset on the NBN Atlas

Welsh Government (2021). Tir Cynnal and Tir Gofal Monitoring and Evaluation Programme: Small Pearl Bordered Fritillary Butterfly survey. Occurrence dataset on the NBN Atlas

Welsh Government (2021). Tir Cynnal and Tir Gofal Monitoring and Evaluation Programme: Whole-farm surveys for yellowhammer and curlew. Occurrence dataset on the NBN Atlas

Welsh Government (2021). Tir Cynnal and Tir Gofal Monitoring and Evaluation Programme: Field-scale winter surveys of yellowhammers. Occurrence dataset on the NBN Atlas

Welsh Government (2021). Tir Cynnal and Tir Gofal Monitoring and Evaluation Programme: Marsh Fritillary Butterfly survey. Occurrence dataset on the NBN Atlas

West Wales Biodiversity Information Centre (2021). INNS Records: All Taxa (West Wales). http://doi.org/10.15468/ydifzd

West Wales Biodiversity Information Centre (2021). NRW Regional Data: all taxa (excluding sensitive species), West Wales. http://doi.org/10.15468/q3d1hl

Wild Surveys (2021). Wild Surveys Common Pip Records. Occurrence dataset on the NBN Atlas

World Museum, National Museums Liverpool (2021). Subset of British spiders in the collections of World Museum, National Museums Liverpool. http://doi.org/10.15468/nvw644

Yorkshire Naturalists' Union (2021). Yorkshire Naturalists Union Marine and Coastal Section Records. Occurrence dataset on the NBN Atlas

Yorkshire Wildlife Trust (2021). Yorkshire Wildlife Trust - Non-sensitive records from all taxonomic groups. http://doi.org/10.15468/2razk5
