# Supplementary material for: Large‐scale correlations between gamebird release and management and animal biodiversity metrics in lowland Great Britain
Source: Ecol Evol. 2023 May 8;13(5):e10059. doi: 10.1002/ece3.10059 (PMC10166649; doi:10.1002/ece3.10059)
Supplement: Supplementary file 3 — Appendix S3 [file ECE3-13-e10059-s003.docx]

**Large-scale correlations between gamebird release and management and animal biodiversity metrics in lowland Great Britain**

Joah Robert Madden^1*^, Rosie Buckley^1^ and Sophia Ratcliffe^2^

**ESM3: Comparison of land cover on Release Grid Squares with all other grid squares in the UK**

We extracted the percentage of each of ten different habitat types using the aggregated land cover data from CEH for each 1km^2^ of Great Britain [Rowland et al. 2017]. The data were filtered to remove all sites with total 0 cover (1km squares found at sea). We then compared the percentages on the 3284 grid squares where gamebird release was reported with the 239,491 grid squares where no releases were reported using a MANOVA, followed by individual tests for each habitat type. The two sets of grid squares differed in all 10 habitat types (ESM Table 1, Fig 1). As expected, RGS contained more arable, broadleaf woodland and improved grassland and less built up, coastal, conifer woodland, montane/bog, semi-natural grassland and salt water cover than an average area in the UK.

|  | Release Grid Square % cover | Other Grid Squares % cover | F_1,241877_ | P |
| --- | --- | --- | --- | --- |
| Broadleaf Woodland | 9.6 | 6.0 | 381.5 | < 0.0001 |
| Coniferous Woodland | 2.8 | 6.2 | 109.0 | < 0.0001 |
| Arable | 41.0 | 22.7 | 1100.6 | < 0.0001 |
| Improved Grassland | 36.5 | 27.3 | 309.0 | < 0.0001 |
| Semi-natural Grassland | 9.5 | 2.9 | 256.5 | < 0.0001 |
| Montane, heath and bog | 1.8 | 14.5 | 568.1 | < 0.0001 |
| Salt water | 0.1 | 1.0 | 36.4 | < 0.0001 |
| Fresh water | 0.5 | 1.1 | 31.1 | < 0.0001 |
| Coastal | 0.6 | 1.8 | 47.9 | < 0.0001 |
| Built up | 3.9 | 7.1 | 98.7 | < 0.0001 |

**ESM Table 1** Comparison of land cover on 1km grid squares where gamebirds are reported as being released and other non-marine grid squares in the UK.


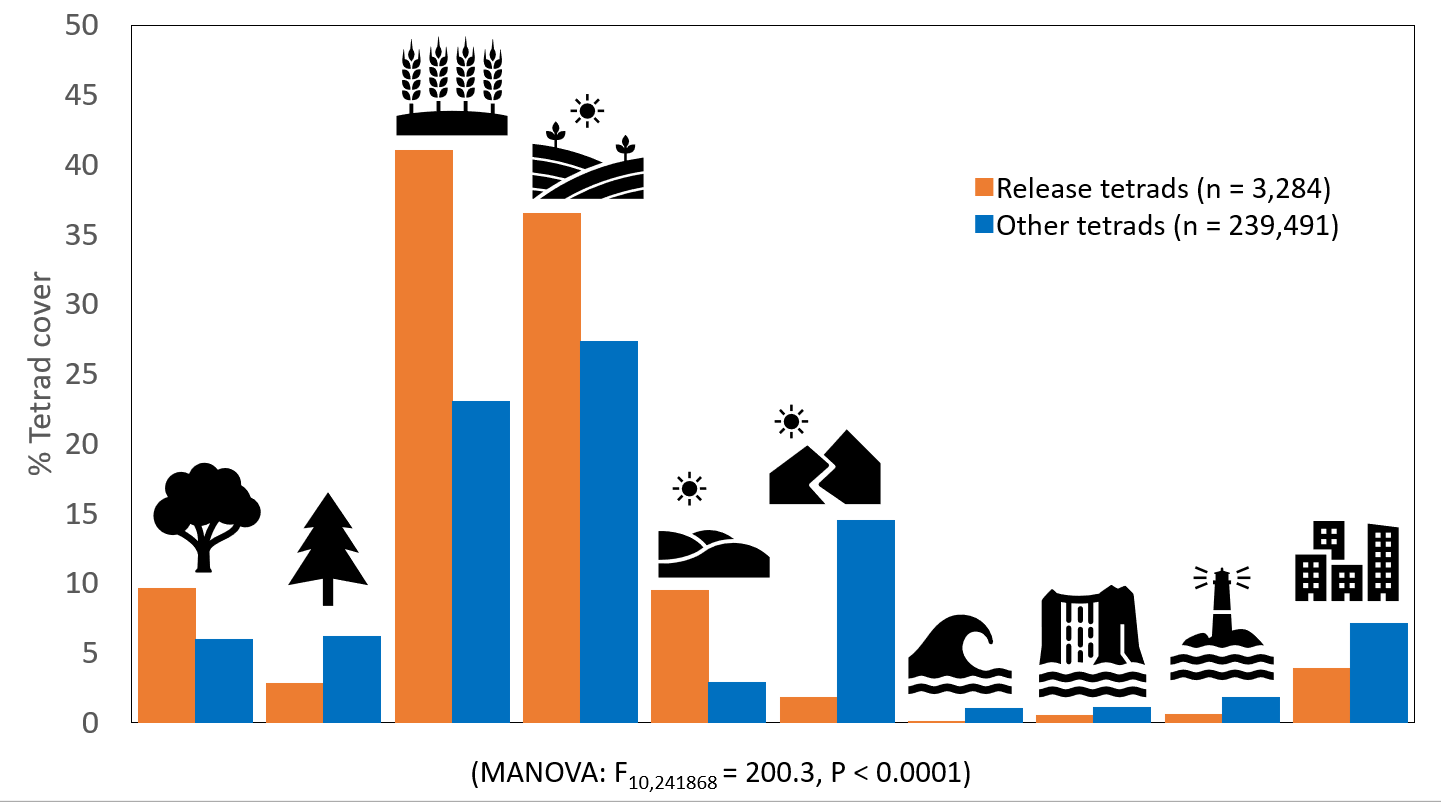


**ESM Figure 1** Comparison of land cover on 1km grid squares where gamebirds are reported as being released and other non-marine grid squares in the UK.
